# Converging Effects of Three Different Endocrine Disrupters on Sox and Pou Gene Expression in Developing Rat Hippocampus: Possible Role of microRNA in Sex Differences

**DOI:** 10.3389/fgene.2021.718796

**Published:** 2021-11-11

**Authors:** Walter Lichtensteiger, Catherine Bassetti-Gaille, Hubert Rehrauer, Jelena Kühn Georgijevic, Jesus A.F. Tresguerres, Margret Schlumpf

**Affiliations:** ^1^ GREEN Tox and Institute of Veterinary Pharmacology and Toxicology, University of Zurich, Zurich, Switzerland; ^2^ Functional Genomics Center, Swiss Federal Institute of Technology and University of Zurich, Zurich, Switzerland; ^3^ Department of Physiology, Universidad Complutense, Madrid, Spain

**Keywords:** endocrine disrupter, development, hippocampus, sox6, pou gene, microRNA, sex difference, transcriptomics

## Abstract

Endocrine disrupting chemicals (EDCs) can impair hippocampus-dependent behaviors in rat offspring and in children. In search for key processes underlying this effect, we compared the transcriptomes of rat hippocampus on postnatal day 6 after gestational and lactational exposure to three different EDCs at doses known to impair development of learning and memory. Aroclor 1254, a commercial PCB mixture (5 mg/kg or 0.5 mg/kg), or bisphenol A (5 mg/kg or 0.5 mg/kg) were administered in chow, chlorpyrifos (3 mg/kg or 1 mg/kg) was injected subcutaneously. Male hippocampus exhibited a common effect of all three chemicals on genes involved in cell-autonomous processes, Sox6, Sox11, Pou2f2/Oct2, and Pou3f2/Brn2, all upregulated at the high dose. Additional genes of the Sox and Pou families were affected by only one or two of the chemicals. Real time RT PCR showed a comparable expression change for bisphenol A also at the lower dose. Female hippocampus exhibited much fewer genes with expression changes (almost none with false discovery rate <0.05), and none of the genes of the Sox and Pou families was affected. Since gene network analyses in male hippocampus suggested a link between Sox6 and miR-24, known to be repressed by activation of ER-alpha and to repress Sox6 in other tissues, this microRNA was measured. miR-24 was downregulated by all chemicals at the high dose in males. Values of Sox6 mRNA and miR-24 were inversely correlated in individual male hippocampus samples, supporting the hypothesis that the change in Sox6 expression resulted from an action of miR-24. In contrast, miR-24 levels remained unchanged in hippocampus of females. A sexually dimorphic response of miR-24 may thus be at the basis of the sex difference in Sox6 expression changes following exposure to the three chemicals. ER-alpha expression was also sex-dependent, but the expression changes did not parallel those of potential downstream genes such as Sox6. Sox6 is known to suppress differentiation of Parvalbumin (Pvalb)-expressing interneurons. Individual Sox6 levels (FPKM) were inversely correlated with levels of Pvalb, but not with markers of Sox6-independent interneuron subpopulations, Nos1 and 5HT3aR. Effects on interneuron development are further suggested, in males, by expression changes of Nrg1 and its receptor Erbb4, controlling interneuron migration. Our study disclosed new types of EDC-responsive morphogenetic genes, and illustrated the potential relevance of microRNAs in sexually dimorphic EDC actions.

## Introduction

The development of cognitive and emotional behaviors represents one of the best documented targets of endocrine disrupting chemicals (EDCs) in humans (Boucher et al., 2009; Rauh et al., 2011; Braun et al., 2017; Ejaredar et al., 2017; Bornehag et al., 2021). Effects on learning and memory and on anxiety have been observed as well in rodent models, for example after perinatal exposure to bisphenol A (Kubo et al., 2001; Ryan and Vandenbergh, 2010; Xu et al., 2010, 2012; Jašarevic et al., 2011; Matsuda et al., 2013; Kumar and Thakur, 2014; Johnson et al., 2016), polychlorinated biphenyls (PCBs) (Gilbert et al., 2000; Roegge et al., 2000; Widholm et al., 2001; Sugawara et al., 2006; Colciago et al., 2009), or phthalates (Zhao et al., 2020). Analogous effects have also been reported for perinatal exposure to the organophosphate pesticide chlorpyrifos (Levin et al., 2001, 2002; Aldridge et al., 2005; Braquenier et al., 2010; Haviland et al., 2010; Turgeman et al., 2011; Chen et al., 2012). Recent data indicate that chlorpyrifos can also interfere with endocrine mechanisms (see below).

From a regulatory perspective, the identification of molecular processes that are common to different types of EDCs with similar adverse effects on brain development, would be of great interest, since knowledge of critical molecular processes could help to design *in vitro* models and molecular markers for introduction into existing *in vivo* OECD test guidelines, and to establish links with the human situation. We compared gene expression patterns in developing rat hippocampus after pre- and postnatal exposure to three EDCs at dose levels known to affect learning and memory in rodents, the PCB mixture, Aroclor 1254 (Aro), bisphenol A (BPA), and the pesticide chlorpyrifos (CPF). Impaired memory function was chosen because it represents a well-documented apical adverse endpoint. Impairment of memory function in rats and mice has been observed after perinatal administration of Aroclor 1254 at oral doses between 1 mg/kg and 10 mg/kg, and after BPA at oral doses between 0.05 mg/kg and 50 mg/kg (References above). BPA doses between 0.0025 mg/kg and 0.02 mg/kg were ineffective (Fujimoto et al., 2006; Ferguson et al., 2012; Nakamura et al., 2012). Chlorpyrifos was effective at subcutaneous doses between 0.1 mg/kg (one report), 1 mg/kg and 5 mg/kg (see Supplemental Table S1). BPA is estrogenic but also interferes with thyroid hormone actions (Rubin, 2011). Aro interacts with thyroid hormones in developing brain (Gauger et al., 2004), and contains estrogenic congeners (Decastro et al., 2006). The acetylcholinesterase inhibitor CPF exerts non-endocrine effects on nerve cells, but has also been found to exhibit estrogenic activity, and to affect thyroid hormone levels in brain (Andersen et al., 2002; Slotkin and Seidler, 2009; Ventura et al., 2012; Slotkin et al., 2013; Hazarika et al., 2020).

Hippocampus is a hormone target during development and postnatal life in animals as well as humans (Ivanova and Beyer, 2000; González et al., 2007; Mogi et al., 2015; Gothié et al., 2017). A special feature of developing hippocampus is that there are two possible sources of sex steroids, the general circulation and local *de novo* synthesis from progesterone (Hojo et al., 2004; Prange-Kiel et al., 2009). The relative role of the two sources has not been clarified, but there is evidence for hippocampal estrogen synthesis during the neonatal period (Amateau et al., 2004; Kretz et al., 2004; Nuñez and McCarthy, 2009). The investigation focused on postnatal day (PND) 6, a stage allowing to investigate a number of different developmental processes, including cell proliferation, differentiation (in particular interneurons), and synaptogenesis (Danglot et al., 2006; Bowers et al., 2010). The early postnatal stage is covered by several OECD test guidelines addressing reproductive and neurodevelopmental toxicity, hence, molecular markers with predictive value could be included into these guidelines.

Transcriptomics analysis revealed analogous effects of the three chemicals on a small number of gene ontology processes related to early neurodevelopment, with sexually dimorphic expression changes of genes involved in cell-autonomous control of development, in particular Sox6, and of downstream genes of Sox6 linked to interneuron development. Sexually dimorphic effects of all three chemicals on an estrogen-regulated microRNA, miR-24, may be involved.

## Methods

### Chemicals

Aroclor 1254 (Lot 124-191-B) was obtained from CromLab S.L. (Barcelona, Spain), bisphenol A (2,2-bis-(4-hydroxyphenyl)-propane, 99%) from Sigma-Aldrich Chemie (Buchs, Switzerland), and chlorpyrifos (chlorpyrifos PESTANAL, analytical standard) from Sigma-Aldrich Quimica (Tres Cantos. Madrid, Spain).

### Animals and Treatment

#### Animals

Long Evans rats were purchased from Janvier Labs (Le Genest Saint Isle Saint Berthevin, France). Experiments were conducted at the Dept. of Endocrinology, Complutense University, Madrid, and approved by the ethical committee of Complutense University. Females (9–10 weeks old) were housed at a 12 h light-dark cycle (lights on 07.00-19.00), 22°C, in groups of 4 during premating, two females with one male (12–14 weeks old) during the 1 week mating period, and in groups of two during pregnancy, being separated on gestational day (GD) 22, 1 day before parturition (GD 1 = day of sperm-positive vaginal smear, GD 23 = postnatal day (PND) 1 = day of birth).

#### Treatment

**Aroclor 1254 (Aro)** and **bisphenol A (BPA)** were administered in the chow at two dose levels, 5.0 mg/kg day (Aro5, BPA5) and 0.5 mg/kg day (Aro0.5, BPA0.5), to female F0 rats 2 weeks before mating, during mating, pregnancy and lactation, until PND 6, with water ad libitum. Both chemicals were admixed to phytoestrogen-low (soy-free) rat chow (IRTA) by the Dept. of Endocrinology (Madrid) and further diluted to the final concentrations, 50 mg/kg chow and 5 mg/kg chow, by IRTA (Institute of Agrifood Research and Technology, Torre Marimon, Barcelona), assuming a daily food intake of 10% of body weight (Historical data of female Long Evans rats in the laboratories of Zurich and Madrid: 8.2 and 10% of body weight, respectively). Feeding controls (CONF) received soy-free chow.

**Chlorpyrifos (CPF)** was dissolved in dimethyl sulfoxide (DMSO) and injected subcutaneously (1 ml DMSO/kg body weight), at 1 mg/kg (CPF1) and 3 mg/kg body weight per day (CPF3), to female F0 rats between GD 2 and GD 20 and again between PND2 and PND 6, in order to avoid possible interactions with parturition. Injection controls (CONSC) received 1 ml DMSO/kg body weight per day. The s. c. route was chosen because the majority of developmental studies with analysis of learning and memory, on which dose selection was based, had used s. c. injection (see Supplementary Table S1).

#### Dose selection

The higher dose level has consistently been found to impair learning/memory in adult rats and mice after pre- and/or early postnatal administration (Supplementary Table S1). In the case of BPA and CPF, a few studies on anxiety were also included in view of the possible role of hippocampus in anxiety. The lower dose of BPA and CPF was effective in some studies, whereas the lower dose of Aro had not been reported to affect learning or anxiety.

### Tissue Preparation and Dissection of Hippocampus

#### Tissue preparation

Offspring were weighed and decapitated at postnatal day (PND) 6. Brain was excised and deep-frozen on dry ice by staff of GREEN Tox assisted by staff of Madrid. Sex was determined by gonadal inspection. Tissues were stored at −80°C before being transferred to Zurich on dry ice and again stored at −80°C. **Time-window**: Brains used for transcriptomics were dissected between 10.30 and 13.20 h. Real time RT PCR was also performed on brain tissue from three Aro5-exposed male pups dissected between 15.45 and 17.50 h. Levels of selected mRNAs analyzed in the project did not differ from those of pups dissected within the time-window.

#### Brain dissection

Dissection of left plus right **dorsal hippocampus** from deep-frozen brain was performed in Zurich. A frontal 1.0 mm thick slice was cut at a level immediately posterior to the optic chiasm in a stainless steel rat brain matrix (Zivic Instruments) cooled to below −10°C by dry ice. From this, a rectangular tissue piece comprising hippocampus plus laterally adjacent cortical tissue was dissected under microscopic control on a Pelletier element (−10°C) (Supplementary Figure S1), transferred into an Eppendorf tube containing 200 μl RNAlater (QIAGEN), and stored at −20°C. Brain dissections were performed in a cold room (4°C). This had been found to result in RIN (RNA integrity number) values for RNA of 8–10, as compared to RIN 5–8 with dissections on cooled plates at room temperature. All dissections were done by the same person (MS), without knowledge of the treatment group.

### Transcriptomics

#### Tissue Homogenization and RNA Extraction

Tissues were transferred to Eppendorf tubes containing RLT buffer, 10 µl ß-mercaptoethanol/1 ml buffer, homogenized, and stored at -80°C. Total RNA was extracted with QIAGEN RNeasy-mini kit, treated with DNase-I (QIAGEN). RIN values ranged between 7.1 and 9.3 in males (Mean ± SD (N): 8.7 ± 0.5 (42)) and between 8.4 and 9.3 in females (9.0 ± 0.2 (24)). Samples with A260/A280 ratio <1.8 were further purified by NucleoSpin® RNA Clean-up XS kit (Macherey-Nagel). RNA samples were stored at −80°C (Males: CONF N = 5, CONSC N = 5, Aro0.5 N = 4, Aro5 N = 6, BPA0.5 N = 4, BPA5 N = 7, CPF1 N = 4, CPF3 N = 7. Females: CONF N = 6, CONSC N = 4, Aro5 N = 5, BPA5 N = 5, CPF3 N = 4).

### RNA-Seq (Illumina RNA Sequencing)

RNA-seq was performed in the Functional Genomics Center (FGCZ) of the ETH Zurich and University of Zurich (J. K. G.) on extracts from dorsal hippocampus of rat pups euthanized between 10.30 and 13.20 h.

#### Library preparation

The quantity and quality of the isolated RNA was determined with a Qubit® (1.0) Fluorometer (Life Technologies, California, United States) and a Bioanalyzer 2100 (Agilent, Waldbronn, Germany). The TruSeq Stranded mRNA Sample Prep Kit (Illumina, Inc, California, United States) was used in the succeeding steps. Briefly, total RNA samples (100 ng) were poly-A selected and then reverse-transcribed into double-stranded cDNA with Actinomycin added during first-strand synthesis. The cDNA samples was fragmented, end-repaired and adenylated before ligation of TruSeq adapters. The adapters contain the index for multiplexing. Fragments containing TruSeq adapters on both ends were selectively enriched with PCR. The quality and quantity of the enriched libraries were validated using Qubit® (1.0) Fluorometer and the Bioanalyzer 2100 (Agilent, Waldbronn, Germany). The product is a smear with an average fragment size of approximately 360 bp. The libraries were normalized to 10 nM in Tris-Cl 10 mM, pH8.5 with 0.1% Tween 20.

#### Cluster Generation and Sequencing

The TruSeq SR Cluster Kit v4-cBot-HS (Illumina, Inc, California, United States) was used for cluster generation using 8 pM of pooled normalized libraries on the cBOT. Sequencing were performed on the Illumina HiSeq 2500 single end 125 bp using the TruSeq SBS Kit v4-HS (Illumina, Inc, California, United States). Adapter sequences shown in Supplementary Table S2.

#### Analysis of RNA-Seq Data

The first part of RNA-seq data analysis was performed by H. R. at the Functional Genomics Center (FGCZ). RNA-seq reads were aligned with the STAR-aligner (Dobin et al., 2013; Love et al., 2014). As reference we used the NCBI genome build for rat Rnor_6.0. Gene expression values were computed with the function featureCounts from the R package Rsubread (Liao et al., 2013). Differential expression was computed using the generalized linear model implemented in the Bioconductor package DESeq2.

Differential expression was computed using ANOVA, followed by Tukey HSD post-hoc test. The two control groups, CONF and CONSC, did not differ significantly from each other. This was further confirmed in a comparison of CONSC and CONF of six target genes by real time RT PCR (Supplementary Figure S2). Thus, a possible difference in the condition of offspring from injection and feeding control groups suggested by body weight, was not reflected in target gene expression in hippocampus. Hence, CONF and CONSC were combined.

Deseq2 data files were further analyzed by enrichment analysis for “significantly affected genes” (different from controls), “differentially affected genes” (different expression in different treatment groups) and “similarly affected genes” (analogous, statistically significant expression changes in the three treatment groups), using MetaCore™ (Thomson Reuters). A group of gene ontology (GO) processes that were similarly affected by the treatments, was further investigated. Extensive gene network analyses (>100) of genes from similarly affected GO processes revealed gene clusters with relevance for neurodevelopment, affected by all three or by two treatments, and a link to a microRNA, miR-24. Literature references documenting links between two genes (listed by MetaCore) were checked in detail in order to assess the reliability of the link. Possible relationships between two genes were further examined by correlation analyses of FPKM (fragments per kilobase per million reads) values of individual hippocampus samples.

### Analysis of Individual mRNAs by Real Time RT PCR

mRNAs of genes of interest identified in network analyses of the transcriptomics data were quantitated by real time RT PCR on extracts from male hippocampus of both dose groups. The males had been euthanized between 10.15 and 13.45, with the exception of three male pups treated with Aroclor 1254, 5 mg/kg, dissected at 15.45, 17.07 and 17.50. In these three pups, the expression levels of the mRNAs studied did not differ from those of the remaining pups. For statistical reasons (see below), two groups of samples were analyzed, samples where transcriptomics had also been done (samples with transcriptomics), and samples where transcriptomics had not been done (samples without transcriptomics). Real time RT PCR was not performed in females, since transcriptomics analysis did not show an expression change in genes of interest.

### Tissue Homogenization and RNA Extraction

Tissues were transferred to Eppendorf tubes containing Lysis Buffer RLT buffer, 10 µl ß-mercaptoethanol/1 ml buffer, homogenized, and homogenates stored at -80°C. Total RNA was extracted with QIAGEN RNeasy-mini kit (Qiagen), treated with DNase-I (QIAGEN). RNA Samples with A260/A280 ratio >1.8 were stored at -80°C.

#### Real Time RT PCR

Real Time RT PCR was performed as previously described (Lichtensteiger et al., 2015). mRNA of hippocampus samples from all eight experimental groups of one sex was analyzed in duplicate and the RNA standard curve in triplicate on 384 wells plates by TaqMan quantitative real-time polymerase chain reaction (CFX384 real-time system, Bio-Rad Laboratories Srl, Milan, Italy). Primers and TaqMan probes were designed with PrimerExpress Software, version: 3.0 (Applied Biosystems), using NCBI Gene Bank and Ensemble database (http://www.ncbi.nlm.nih.gov, http://www.ensembl.org), and synthesized by Microsynth (Balgach, Switzerland) (List see Supplementary Table S3). Since investigations in a foregoing study (Lichtensteiger et al., 2015) had shown that expression of 18 possible reference genes was affected by treatment with endocrine active chemicals, quantitation of target mRNAs was based on the standard curve method. Total RNA, containing 80% ribosomal RNA, was considered to represent a stable reference, because expression of two of the four ribosomal RNAs (Rn18, Rn28) had previously been found to be unchanged (Lichtensteiger et al., 2015).

#### Analysis of Real Time RT PCR Data

mRNA levels of individual samples were expressed as percentage of the mean of the corresponding control group. One-way analysis of variance (ANOVA) was performed for each mRNA species, followed by Dunnett’s multiple comparisons test (GraphPad Prism 7). Three ANOVA analyses were conducted for each mRNA species: Samples also used in transcriptomics analysis, samples not used in transcriptomics analysis, and both groups combined (Males total N: CONSC = 8, CONF = 10, Aro0.5 = 11, Aro5 = 10, BPA0.5 = 10, BPA5 = 12, CPF1 = 11, CPF3 = 11). The group of samples **without transcriptomics analysis** represents a statistical sample which is independent of transcriptomics analysis and hence, allows to control the transcriptomics data. This is particularly important for genes with a false discovery rate in transcriptomics of Fdr >0.05. In the present investigation, this was only the case for Pou2f1/Oct1 with Fdr = 2.14E-01 for bisphenol A, and Fdr = 1.12E-01 for Aroclor 1254. Fdr values of all other genes of interest ranged between 2E-02 and 9E-07. Figures show results with samples not used in transcriptomics analysis (1 per litter).

## Determination of microRNA-24 (miR-24-3p)

### Tissue Homogenization and microRNA Extraction

miR-24-3p analysis was limited to the high-dose groups because there was not enough tissue material left for analysis of the low-dose groups. Frozen tissues were homogenized in Lysis Buffer RLT buffer (Qiagen) and miRNAs were extracted using mirVana miRNA Isolation Kit (Ambion) following the manufacturer’s protocol. The majority of extracts was obtained from hippocampus samples also extracted for real time RT PCR.

#### Determination of miR-24-3p

miR-24-3p originates from two gene clusters, miR 23b, miR 27b, miR-24-1 (rat chromosome 17), and miR-23a, miR-27a, miR-24-2 (rat chromosome 19), but the mature sequence of miR-24-3p-1 and miR-24-3p-2 is the same, miR-24-3p (Chhabra et al., 2010; Marchetti et al., 2020). miR-24-3p is documented for a number of species. Rat miRBase ID is rno-miR-24-3p, miRBase Accession Number MIMAT0000794, the mature miRNA Sequence UGG​CUC​AGU​UCA​GCA​GGA​ACA​G.

Poly(A) tailing, ligation, reverse transcription and amplification reactions were performed with the TaqMan Advanced miRNA cDNA Synthesis Kit (Applied Biosystems/ThermoFisher Scientific), using 10 ng total RNA input. qRT-PCR experiments were performed in duplicate on 384-well plates with TaqMan Fast Advanced Master Mix (Applied Biosystems/ThermoFisher Scientific) and TaqMan primer/probe (TaqMan Advanced miRNA Assay, Assay ID rno481011_mir, Applied Biosystems/ThermoFisher Scientific). PCR amplifications were performed on CFX384 real-time system (Bio-Rad Laboratories Srl, Milan, Italy). Quantitation of target mRNAs was based on the standard curve method (see above).

#### Analysis of miR-24-3p Data

microRNA levels of individual samples were expressed as percentage of the mean of the corresponding control group. One-way analysis of variance (ANOVA) was performed for each microRNA, followed by Dunnett’s multiple comparisons test (GraphPad Prism 7).

## Results

### General Developmental Endpoints

Male and female offspring born to control dams receiving subcutaneous injections of vehicle showed lower body weights than offspring of the feeding control (Table 1). Yet, body weights as well as total number of pups exposed to the three chemicals were at the level of the corresponding, feeding or injection controls, indicating that the chemicals did not affect body weight or total number of pups. There also were no significant differences in sex ratio.

**TABLE 1:** Body weight and number of rat offspring at postnatal day 6 (litter mean).

	Body weight (g)		Number of pups per litter		Sex ratio
	Males	Females	Males	Females	Litter mean ± SD (Number of litters)
	Litter mean ± SD (Number of litters)	Litter mean ± SD (Number of litters)	Litter mean ± SD (Number of litters)	Litter mean ± SD (Number of litters)	
Injection Control	9.65 ± 1.37 ^a^ (8)	9.10 ± 1.32 ^a^ (8)	3.00 ± 1.77 ^b^ (8)	4.38 ± 3.07 ^b^ (8)	43.00 ± 24.06 (8)
Chlorpyrifos, 1 mg/kg	9.80 ± 2.02 (10)	9.24 ± 1.96 (11)	4.00 ± 2.45 (10)	4.00 ± 1.79 (11)	46.93 ± 20.36 (11)
Chlorpyrifos, 3 mg/kg	9.29 ± 2.39 (7)	8.60 ± 2.36 (7)	3.00 ± 1.29 (7)	3.86 ± 1.35 (7)	43.69 ± 16.55 (7)
Feeding Control	12.44 ± 1.44 (10)	11.79 ± 1.41 (10)	4.70 ± 1.95 (10)	5.50 ± 2.55 (10)	47.47 ± 15.73 (10)
Bisphenol A, 0.5 mg/kg	10.88 ± 0.78 (8)	10.59 ± 0.92 (8)	5.00 ± 1.69 (8)	4.25 ± 1.91 (8)	54.31 ± 12.11 (8)
Bisphenol A, 5 mg/kg	11.36 ± 1.23 (11)	11.04 ± 1.30 (11)	4.91 ± 2.30 (11)	5.27 ± 2.24 (11)	48.21 ± 15.30 (11)
Aroclor 1254, 0.5 mg/kg	11.95 ± 1.47 (9)	11.53 ± 1.54 (10)	5.89 ± 3.06 (9)	5.40 ± 1.58 (10)	45.55 ± 21.43 (10)
Aroclor 1254, 5 mg/kkg	11.62 ± 1.31 (8)	11.00 ± 1.45 (8)	5.63 ± 2.83 (8)	6.25 ± 1.49 (8)	44.46 ± 14.85 (8)

a Injection Control vs Feeding Control, males *p* < 0.001, females *p* < 0.001.

b Injection Control vs Feeding Control, males *p* < 0.001, females *p* < 0.01. No differences between treated groups and corresponding controls. ANOVA with Šídák’s multiple comparisons test.

### Gene Expression in Hippocampus of Male and Female Rat Offspring on Postnatal Day 6

Analyses were performed on dorsal hippocampus (plus a small part of laterally adjacent neocortical tissue, Supplementary Figure S1) on postnatal day (PND) 6, when pups were still exposed to the chemicals. In the high-dose groups, the dose range consistently linked with alterations of hippocampus-dependent behaviors (Aroclor 1254, 5 mg/kg, bisphenol A, 5 mg/kg, chlorpyrifos, 3 mg/kg), transcriptomics analysis in hippocampus of male offspring revealed significant changes in gene expression (Table 2). ANOVA of hippocampus of male offspring exposed to a 10 times lower dose (Aroclor 1254, 0.5 mg/kg, bisphenol A, 0.5 mg/kg) or to a three times lower dose (chlorpyrifos, 1 mg/kg) yielded an uncertain result, with high false discovery rates (Fdr) in the great majority of genes (Table 2). Real time RT PCR, where a larger number of samples was studied, showed signficant changes in some target genes also in the low-dose groups (see below). Transcriptomics analysis of high-dose groups in females showed a much lower number of significantly affected genes, and the great majority of genes showed a high false discovery rate, suggesting high interindividual variability (Table 2, Table 4). Methodological aspects, RNA quality and RNA-seq conditions were found to be very good, at the level of the analysis of male samples. The RNA-seq assay of female hippocampus was repeated, with the same result. Since the number of samples in the various experimental groups was similar to that of males (see Methods), expression changes of a magnitude comparable to the high-dose groups of males should have been detectable.

**TABLE 2:** Number of genes with significant change of expression rate in transcriptomics analyses of rat hippocampus at postnatal day 6, according to *p* value and false discovery rate (Fdr).

	Higher dose level		Lower dose level
	Males	Females	Males ^c^
Aroclor 1254	5 mg/kg (Aro5)	5 mg/kg (Aro5)	0.5 mg/kg (Aro0.5)
*p* < 0.05^a^	4,240	1,092	3,327
*p* < 0.05 as well as Fdr <0.05^b^	2725	7	287
Bisphenol A	5 mg/kg (BPA5)	5 mg/kg (BPA5)	0.5 mg/kg (BPA0.5)
*p* < 0.05	2447	898	1,103
*p* < 0.05 as well as Fdr <0.05	751	4	21
Chlorpyrifos	3 mg/kg (CPF3)	3 mg/kg (CPF3)	1 mg/kg (CPF1)
*p* < 0.05	3,432	1,261	0
*p* < 0.05 as well as Fdr <0.05	1,548	0	0

a All values with *p* < 0.05, including values with Fdr >0.05.

b Number of values fulfilling both criteria, *p* < 0.05 as well as Fdr <0.05.

c No RNA-seq assay was conducted with lower dose female groups.

**FIGURE 1:**
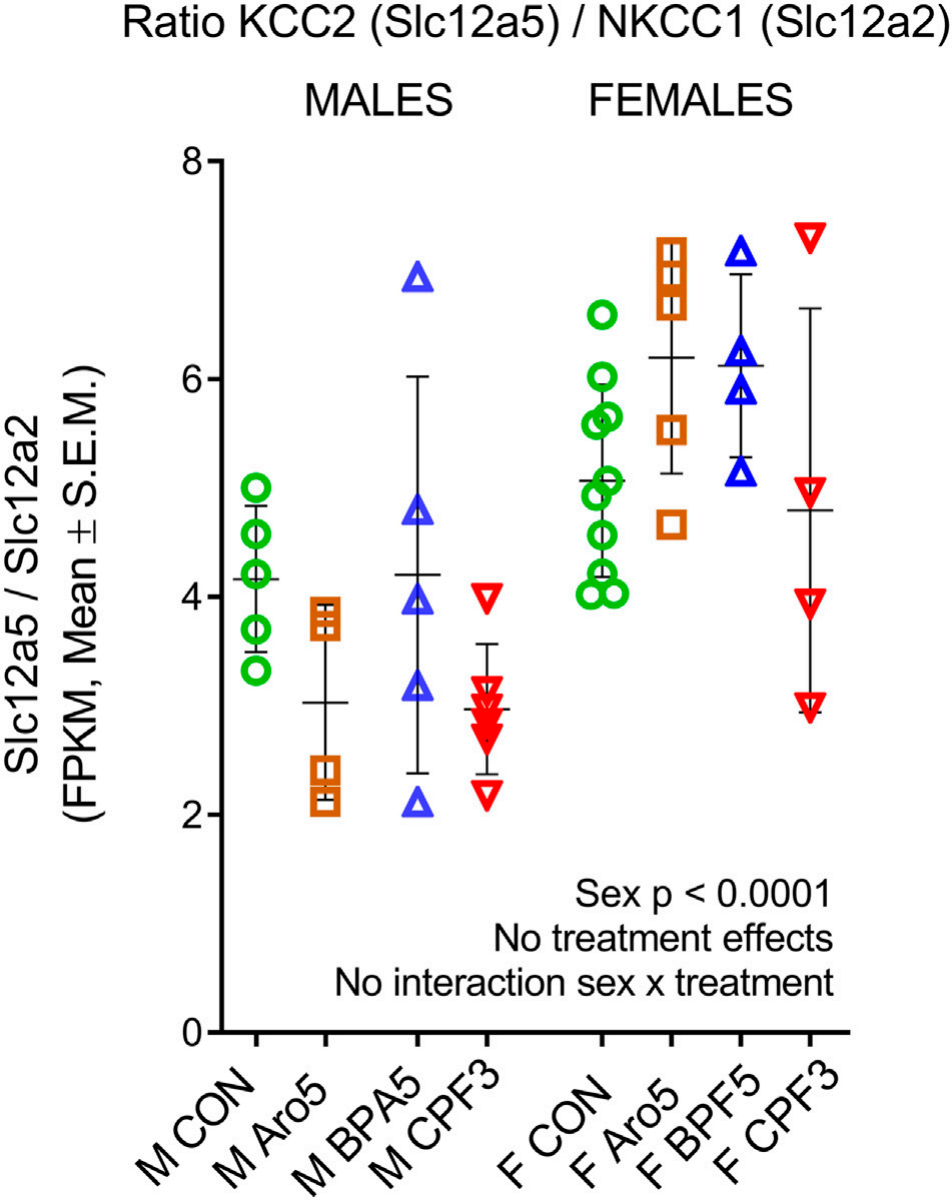
Epression ratio of potassium-chloride-co-transporter KCC2 (Slc12a5), neuron-specific chloride ion extruder, to sodium-potassium-chloride-co-transporter NKCC1 (Slc12a2), increasing intracellular chloride concentration, in individual hippocampus samples of male and female rat offspring at postnatal day 6. RNAseq data, ratio of FPKM (fragments per kilobase per million reads), mean ± SD and individual samples (M, male, F, female). Two way ANOVA, sex difference (*p* < 0001), but no treatment effects and no sex × treatment interaction.

The developmental switch of GABAergic transmission from excitation to inhibition, characterized by an increase in the expression ratio of the Cl-extruder KCC2, a neuron-specific potassium-chloride-co-transporter, relative to NKCC1, the sodium-potassium-chloride-co-transporter increasing intracellular Cl-concentration, takes place in hippocampus at the end of the first postnatal week (Watanabe and Fukuda, 2015). In line with previous findings (Nuñez and McCarthy, 2009), the KCC2/NKCC1 ratio (expressed as fragments per kilobase per million reads) was higher in female hippocampus (Figure 1, *p* < 0.0001), however, this general developmental trait was not significantly influenced by the chemicals.

### Gen Ontology Processes and Gene Networks Related to Neurodevelopment

Enrichment analysis in **male hippocampus** (significance level *p* < 0.01, effect threshold Thr (Log2 ratio) = 0.5 ≅ 141% of control level) focused on male offspring exposed to the higher dose levels, since ANOVA was essentially negative for the lower dose groups. It revealed a number of significantly affected gene ontology (GO) processes related to brain development and function (Supplementary Table S4). Since all three treatments impair development of learning and memory in rodents, we reasoned that it should be possible to identify **GO processes in hippocampus that were similarly affected by all three chemicals**, and similarities in gene expression patterns. An enrichment analysis for similarity of changes (*p* < 0.01, Thr = 0.5, Supplementary Figure S3) identified several GO processes related to development of hippocampus and cerebral cortex, with −log (*p* value) between 3 and 5 for all three treatments (Table 3, Supplementary Table S5 with all significantly affected genes). Interestingly, these GO processes control early developmental processes: Neuroepithelial cell differentiation, regulation of stem cell proliferation, regulation of neuroblast proliferation, positive regulation of neuroblast proliferation, positive regulation of neural precursor cell proliferation, cerebral cortex radially oriented cell migration, gliogenesis, oligodendrocyte development. In contrast, enrichment analysis for similarity of processes in **female hippocampus** (*p* < 0.01, Thr (Log2 ratio) = 0.1) did not show a clear-cut focus on GO processes related to neurodevelopment. Only two very general terms, “generation of neurons” (38/100) and “nervous system development” (40/100) reached *p* values below 1E-05 (Supplementary Figure S4). Moreover, there was little overlap of significantly affected genes.

**TABLE 3:** GO processes similarly affected by all three treatments in male hippocampus, with genes selected for network analyses^a^ ^,^ ^b^.

GO process with −log (*p* value) range of the 3 treatment groups	Treat-ment ^c^	Gli3 p value	Sox6 p value	Sox11 p value	Pou3f2 p value	Tcf7l2 Tcf-4 p value	Fzd3 p value	Notch2 p value
Neuroepithelial cell differentiation −log (*p* value) 4.5 to 5	Aro5	1.01E-11		7.72E-05		2.87E-06		1.02E-06
	BPA5	4.29E-06				1.89E-04		
	CPF3	1.51E-06		1.94E-03		1.13E-05		1.64E-05
Regulation of stem cell proliferation −log (*p* value) 4.5 to 5	Aro5	1.01E-11		7.72E-05			4.97E-13	1.02E-06
	BPA5	4.29E-06		3.15E-02			1.45E-05	1.41E-03
	CPF3	1.51E-06					3.09E-06	
Regulation of neuroblast proliferation −log (*p* value) · 4	Aro5	1.01E-11					4.97E-13	1.02E-06
	BPA5	4.29E-06					1.45E-05	
	CPF3	1.51E-06					3.09E-06	1.64E-05
Positive regulation of neuroblast proliferation −log (*p* value) 3.5	Aro5	1.01E-11					4.97E-13	1.02E-06
	BPA5	4.29E-06					1.45E-05	
	CPF3	1.51E-06					3.09E-06	1.64E-05
Positive regulation of neural precursor cell proliferation −log (*p* value) = 2.5 to 3	Aro5	1.01E-11					4.97E-13	1.02E-06
	BPA5	4.29E-06					1.45E-05	
	CPF3	1.51E-06					3.09E-06	1.64E-05
Cerebral cortex radially oriented cell migration −log (*p* value) 3	Aro5	1.01E-11			6.79E-05			
	BPA5	4.29E-06			3.07E-04			
	CPF3	1.51E-06						
Gliogenesis −log (*p* value) ≅ 4.5 to 5	Aro5	1.01E-11	1.89E-06	7.72E-05	6.79E-05	2.87E-06		1.02E-06
	BPA5	4.29E-06	5.86E-04	3.15E-02	3.07E-04	1.89E-04		1.41E-03
	CPF3	1.51E-06	2.11E-04		2.45E-04	1.13E-05		
Oligodendrocyte development −log (*p* value) ≅ 3	Aro5			7.72E-05		2.87E-06		
	BPA5					1.89E-04		
	CPF3			1.94E-03		1.13E-05		

a Complete list of genes associated with similarly affected GO processes in Supplementary Table S5.

b List of all significantly affected GO processes in Supplementary Table S4.

c Aro5: Aroclor 1254, 5 mg/kg; BPA5: bisphenol A, 5 mg/kg; CPF3: chlorpyrifos, 3 mg/kg.

Gli3 (GLI family zinc finger 3); Sox6 (SRY-box transcription factor 6); Sox11 (SRY-box transcription factor 11); Pou3f2/Brn2 (POU class 3 homeobox 2, Pou = Pituitary-specific Pit-1/Octamer transcription factor proteins/neural Unc-86 transcription factor); Tcf7L2 (Transcription factor 7 like 2, TCF4); Fzd (Frizzled class receptor 3); Notch2 (Notch receptor 2).

The group of similarly affected GO processes in male hippocampus shows several genes involved in **cell-autonomous regulation of developmental processes**, Gli6, Sox6, Sox11, and Pou3f2 (Brn2), the non-canonical Wnt receptor, Fdz3, and the transcription factor Tcf7L2 (Table 3). Possible links with endocrine actions were analyzed for these genes as well as for Nrg1 and its receptor Erbb4, previously found to be targeted by EDC mixtures (Lichtensteiger et al., 2015). Gene networks (n = 154) with information on transcriptional regulation, receptor binding, and phosphorylation, were constructed in MetaCore for each of these genes in combination with sex steroid receptors (Esr1, Esr2, AR), thyroid hormone receptors (TRa, TRb1), arylhydrocarbon receptor (AhR), and components of signaling pathways, with significance level *p* < 0.05 and Thr (Log ratio) = 0.5 (sometimes 0.3). Attention was given to possible links with miRs.

### Sox and Pou Domain Genes Involved in Cell-Autonomous Regulation of Development

**FIGURE 2:**
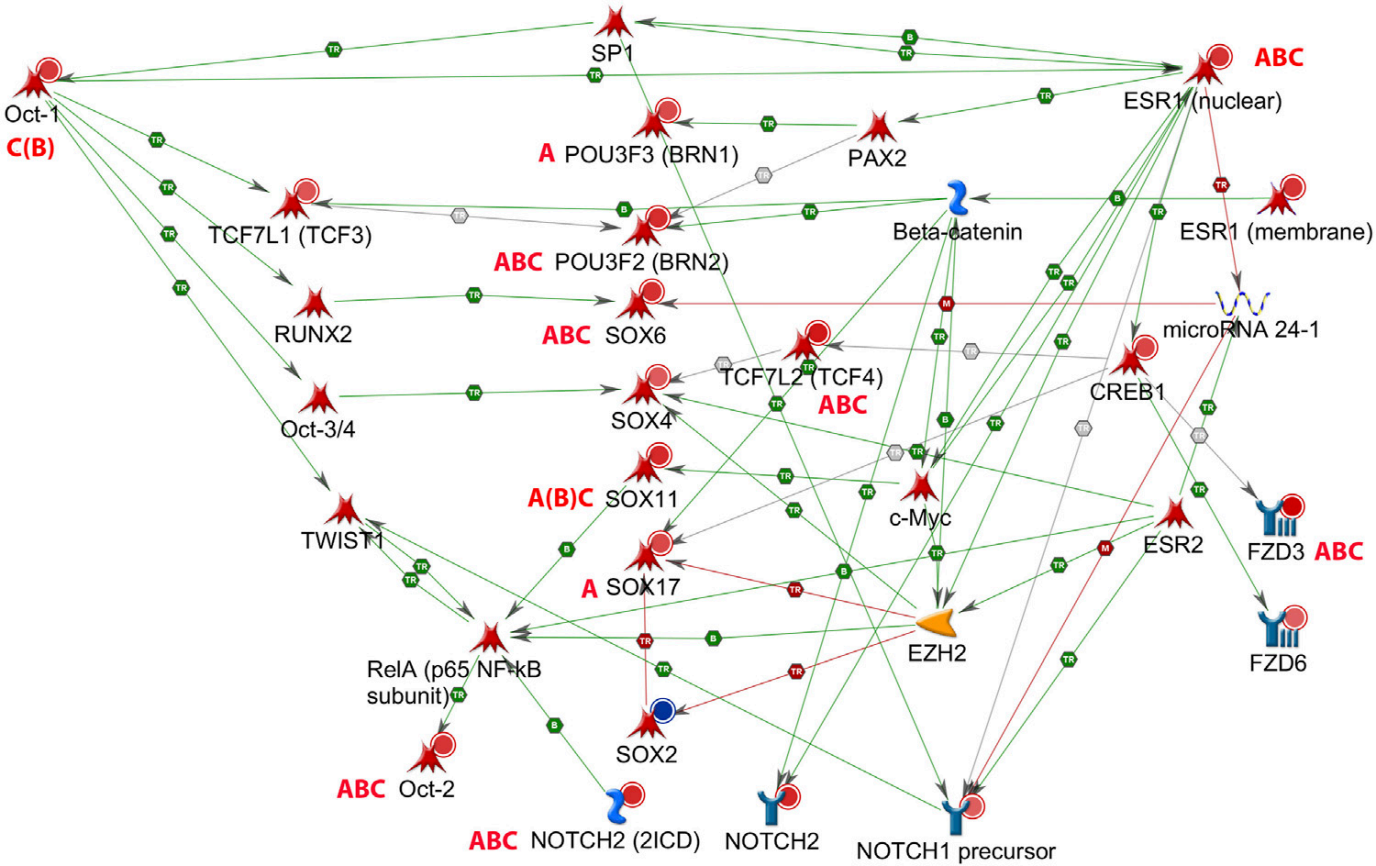
Gene network of male rat hippocampus at postnatal day (PND) 6, with estrogen receptor alpha (Esr1). Up-regulation (red circles) or down-regulation (blue circle) of hippocampal genes exposed to Aroclor 1254 (5 mg/kg, A), bisphenol A (5 mg/kg, B), or chlorpyrifos (3 mg/kg, C) (see Table 4). Pou2f1/Oct1 and Sox11 exhibited a false discovery rate >0.05 after bisphenol A, but the effect was confirmed by real time RT PCR (Figure 3). Literature data indicate a possible inhibitory action of Esr1 on transcription of miR-24-3p, and of miR-24-3p on transcription of Sox6 (MetaCore, Shortest Paths network. TR: transcription, B: binding, M: miR binding, green: activation, red: inhibition).

**FIGURE 3:**
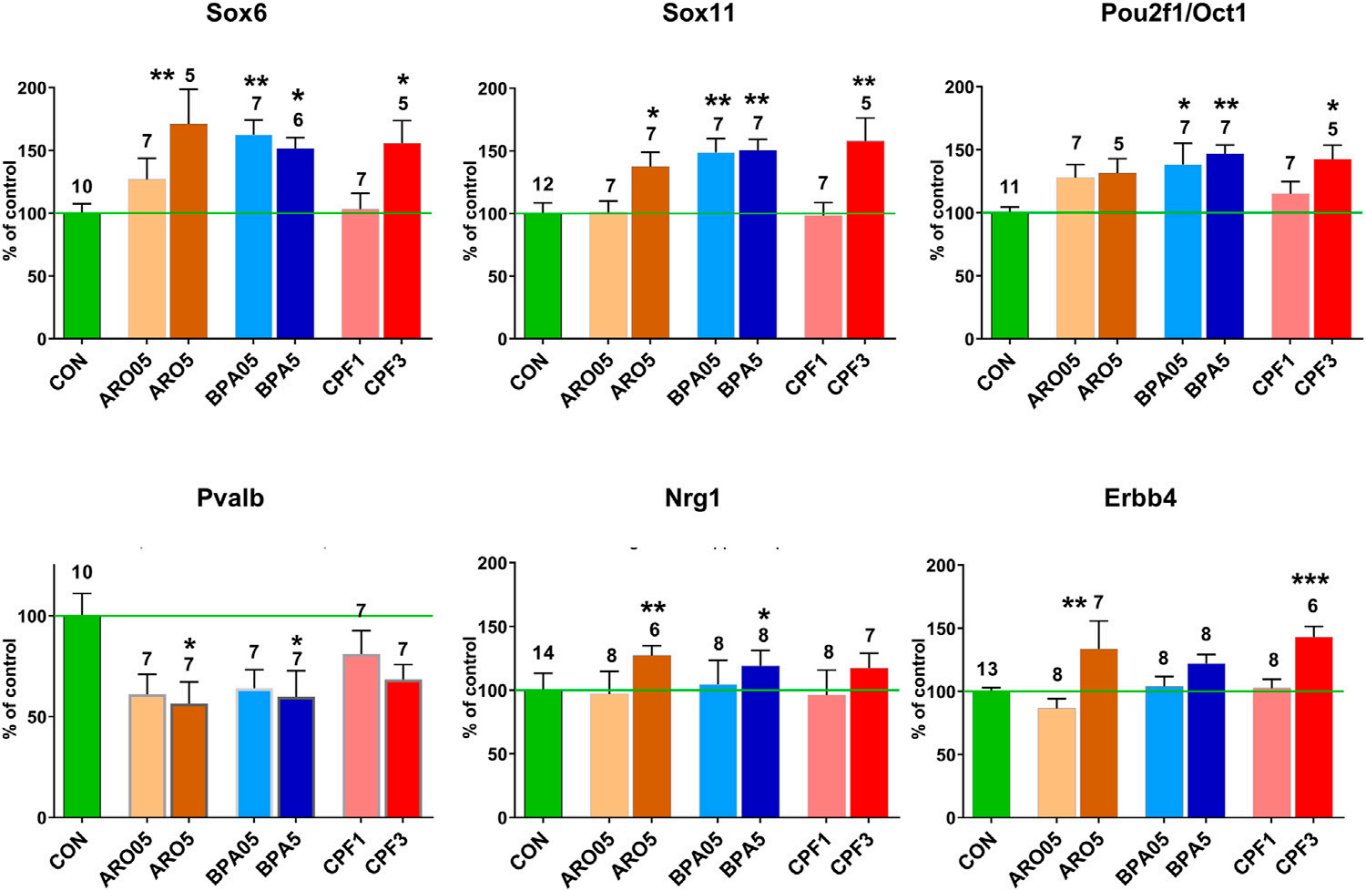
mRNA levels in male hippocampus at postnatal day 6 after pre- and postnatal administration of Aroclor 1254 (ARO0.5: 0.5 mg/kg, ARO5: 5 mg/kg), bisphenol A (BPA0.5: 0.5 mg/kg, BPA5: 5 mg/kg), or chlorpyrifos (CPF1: 1 mg/kg, CPF3: 3 mg/kg). Quantitative RT PCR of hippocampus samples not used for transcriptomics. Mean ± SEM, number of samples (one/litter). Difference from control: *p < 0.05, **p < 0.01, ***p < 0.001.

Network analyses based on the aforementioned genes revealed a possible involvement of a number of genes of the Sox and Pou families in male hippocampus (Figure 2). **Sox6, Sox11, Pou2f2/Oct2**, and **Pou3f2/Brn2** stand out as being similarly affected by all three chemicals, besides Gli3 (Table 4). Expression of Sox5, which interacts with Sox6 during certain developmental phases (Azim et al., 2009; Batista-Brito et al., 2009), was unchanged. Some other Sox and Pou genes were significantly affected by only one or two chemicals. Expression of these genes in female hippocampus was at control level (Supplementary Table S6). In an additional set of hippocampus samples of male offspring from the same litters but not used for transcriptomics, levels of Sox6, Sox11, and Pou2f1/Oct1 mRNA were determined by real time RT PCR (Figure 3). Pou2f1/Oct1 was chosen because transcriptomics results were uncertain with high Fdr values for Aroclor 1254 and bisphenol A (5 mgkg). This analysis confirmed the effects of all three chemicals at the high dose level on Sox6, Sox11, and of bisphenol A and chlorpyrifos on Pou2f1/Oct1. Real time RT PCR revealed significant effects of bisphenol A on all three genes also at the lower dose level (Figure 3), possibly because this analysis was based on a larger sample size than transcriptomics. The magnitude of expression changes detected by RNA-seq and real time RT PCR was very similar (Supplementary Table S7).

**TABLE 4:** Male hippocampus at postnatal day 6: Expression of similarly affected genes (transcriptomics)^a^.

Gene	Aroclor 1254 5 mg/kg			Bisphenol A 5 mg/kg			Chlorpyrifos 3 mg/kg		
	Log ratio	% CON	*p* Value *Fdr* *^b^*	Log ratio	% CON	*p* Value *Fdr* *^b^*	Log ratio	% CON	*p* Value *Fdr* *^b^*
**Genes involved in cell-autonomic regulation of development**									
Gli3	0.9904	198.67	1.01E-11 *5.00E-09*	0.6537	157.32	4.29E-06 *1.32E-03*	0.6665	158.72	1.51E-06 *1.94E-04*
Pou2f1 Oct1	0.3510	127.54	3.07E-02 *1.12E-01*	0.3375	126.36	3.38E-02 *2.14E-01* *^c^*	0.5499	146.40	4.10E-04 *7.96E-03*
Pou2f2 Oct2	0.5813	149.62	9.99E-05 *1.39E-03*	0.4443	136.07	2.23E-03 *4.34E-02*	0.4694	138.45	9.21E-04 *1.39E-02*
Pou3f2 Brn2	0.6479	156.69	6.79E-05 *1.02E-03*	0.5844	149.94	2.45E-04 *1.27E-02*	0.5639	147.83	3.07E-04 *6.56E-03*
Pou3f3 Brn1	0.4127	133.12	1.00E-02 *4.94E-02*	0.1504	110.99	3.38E-01 *6.60E-01*	0.3403	126.60	2.66E-02 *1.30E-01*
Sox2	−0.2980	81.34	4.17E-03 *2.55E-02*	−0.1130	92.47	2.54E-01 *5.83E-01*	−0.2039	86.82	3.29E-02 *1.47E-01*
Sox4	0.1495	110.92	2.49E-01 *4.57E-01*	0.1159	108.37	3.53E-01 *6.75E-01*	0.2748	120.98	2.29E-02 *1.18E-01*
Sox5	0.1749	112.89	1.28E-01 3.00E-01	0.0700	104.97	5.25E-01 7.91E-01	0.1612	111.82	1.29E-01 3.36E-01
Sox6	0.6096	152.58	1.89E-06 *6.00E-05*	0.4242	134.18	5.86E-04 *2.09E-02*	0.4430	135.94	2.11E-04 *5.09E-03*
Sox11	0.6825	160.49	7.72E-05 *1.14E-03*	0.3682	129.07	3.15E-02 *2.06E-01* *^c^*	0.5251	143.90	1.94E-03 *2.35E-02*
Sox17	0.4257	134.32	2.88E-03 *1.92E-02*	0.2556	119.38	6.61E-02 *3.06E-01*	0.2934	122.55	3.00E-02 *1.39E-01*
**Genes encoding for growth factors and receptors of transcellular signaling pathways**									
Nrg1	0.5275	144.14	4.91E-05 *7.92E-04*	0.4759	139.08	1.46E-04 *9.59E-03*	0.4038	132.30	9.14E-04 *1.38E-02*
Erbb4	0.9423	192.16	7.22E-09 *8.85E-07*	0.4495	136.56	5.04E-03 *6.98E-02*	0.7273	165.55	3.49E-06 *3.12E-04*
Efnb2	0.7238	165.15	5.08E-06 *1.33E-04*	0.3496	127.42	2.44E-02 *1.77E-01*	0.5654	147.98	1.93E-04 *4.80E-03*
Eph4	0.5849	149.99	3.51E-06 *9.84E-05*	0.3810	130.22	1.67E-03 *3.72E-02*	0.5537	146.78	2.33E-06 *2.49E-04*
Fzd3	1.0070	200.97	4.97E-13 *4.74E-10*	0.5888	150.40	1.45E-05 *2.51E-03*	0.6152	153.18	3.09E-06 *2.90E-04*
Tcf7L2	0.8103	175.36	2.87E-06 *8.36E-05*	0.6416	156.01	1.89E-04 *1.09E-02*	0.7473	167.86	1.13E-05 *6.97E-04*
Notch2	0.7017	162.64	1.02E-06 *3.78E-05*	0.4444	136.07	1.41E-03 *3.42E-02*	0.5827	149.76	1.64E-05 *8.91E-04*

a Values of female offspring in Supplementary Table S6

b Fdr: False discovery rate.

c Significant in real time RT PCR of samples not used for transcriptomics.

Efnb2: Ephrin B2, Eph4: ephrin type-A receptor 4

### Genes Encoding for Growth Factors and Receptors of Transcellular Signaling Pathways

In male hippocampus, neuregulin 1 (Nrg1), which controls migration and other aspects of development of cortical interneurons (Mei and Nave, 2014), was found to be up-regulated in transcriptomics by all three treatments and in real time RT PCR by the higher dose levels of Aroclor 1254 and bisphenol A (Table 4; Figure 3). Its receptor Erbb4, expressed in hippocampal interneurons (Mei and Nave, 2014), was up-regulated by the higher doses of Aroclor 1254 and chlorpyrifos, as shown by both methods of analysis. In female hippocampus, a slight reduction of Erbb4 mRNA to 88.2% CON by 5 mg/kg of Aroclor 1254 is uncertain because of an increased Fdr. Nrg1 expression was unchanged (Supplementary Table S6). Interestingly, transcriptomics did not reveal expression changes of a number of growth factors and their receptors in male hippocampus, including BDNF, GDNF, EGF, IGF, NGF, and Vegfa. Fgf14 was upregulated by all three treatments and Fgf12 by Aroclor 1254 (data not shown). The GO analyses also showed significant effects on Fzd3, involved in non-canonical Wnt signaling, and Notch2 (Table 3), but since no additional changes in these pathways or related genes were observed in network analyses, they were not pursued further. Transcriptomics of female hippocampus did not reveal significant expression changes of the aforementioned genes.

### miR-24 and Sexually Dimorphic Regulation of Sox6

**FIGURE 4:**
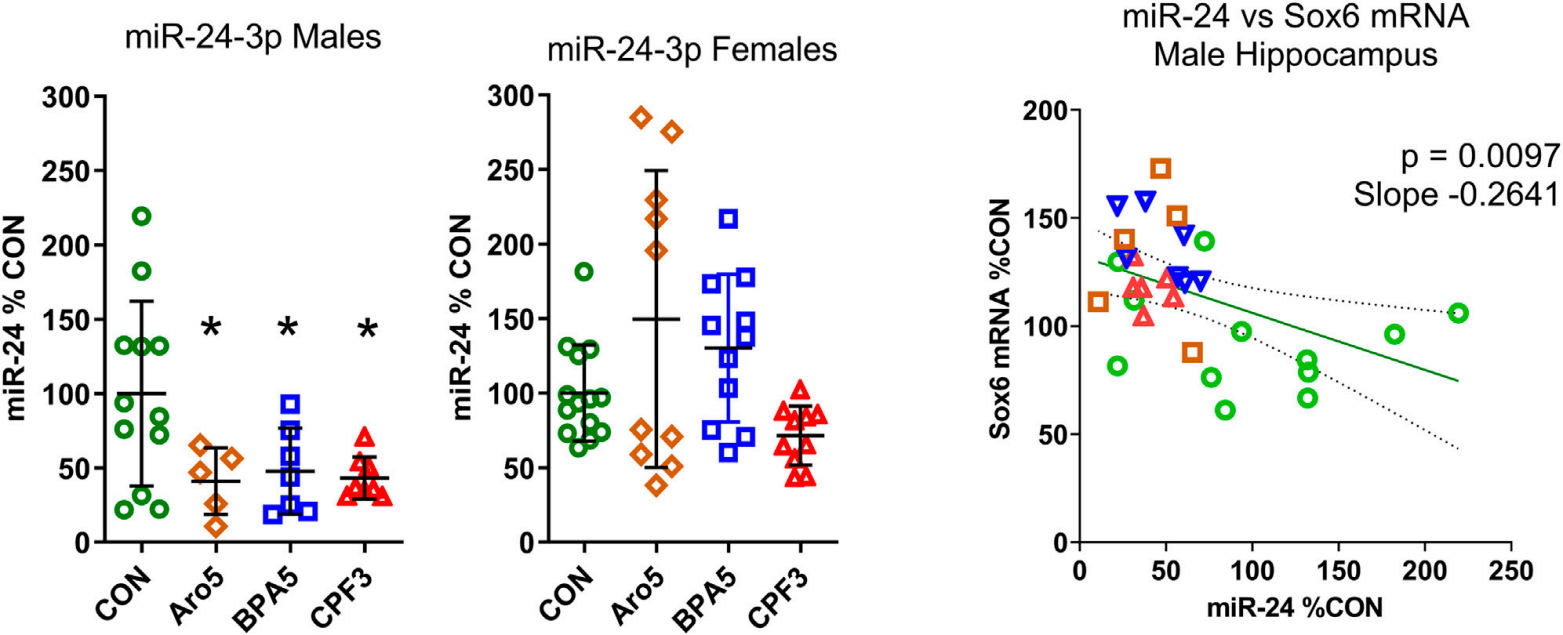
A. Level of miR-24-3p in hippocampus of male and female rat offspring at postnatal day 6 after pre- and postnatal administration of Aroclor 1254 (5 mg/kg, Aro5), bisphenol A (5 mg/kg, BPA5), or chlorpyrifos (3 mg/kg, CPF3). Quantitative RT PCR, individual values with mean ± S.D, *p < 0.05 different from control group. CON: controls, ARO5: Aroclor 1254, 5 mg/kg, BPA5: bisphenol A, 5 mg/kg, CPF3: chlorpyrifos 3 mg/kg. B. Relationship between miR-24-3p (% of control) and Sox6 mRNA (% of control, real time RT PCR) in individual hippocampus samples of male offspring. Inverse correlation, p = 0.0097, r = −0.4645, slope = −0.264. Green symbols: control, brown: Aroclor 1254 5 mg/kg, blue: bisphenol A 5 mg/kg, red: chlorpyrifos 3 mgkg.

It is not known what might be the basis of the sex difference in the responsiveness of the Sox and Pou genes to the three chemicals. In the case of Sox6, our gene network analyses suggested a possible link between ER-alpha (Esr1), miR-24, and Sox6 (Figure 2). ER-alpha-mediated repression of miR-24 transcription, and repression of Sox6 transcripton by miR-24, has been demonstrated in non-neural human cell lines and mouse pancreatic beta cells (Maillot et al., 2009; Melkman-Zehavi et al., 2011; Ferraro et al., 2012; Klinge, 2012). miR-24 has previously been detected in rat hippocampus (Liu et al., 2016). It is derived from two miR clusters which may be differentially regulated, but the mature miR-24-3p sequence derived from the two clusters is the same (Chhabra et al., 2010). miR-24-3p was significantly down-regulated in hippocampus of male offspring by all three chemicals administered at the higher dose level, whereas no significant expression change was observed in hippocampus of females (Figure 4). A significant inverse relationship existed between miR-24-3p levels and Sox6 mRNA levels (real time RT PCR data) in individual hippocampus samples of male offspring (Figure 4).

**FIGURE 5:**
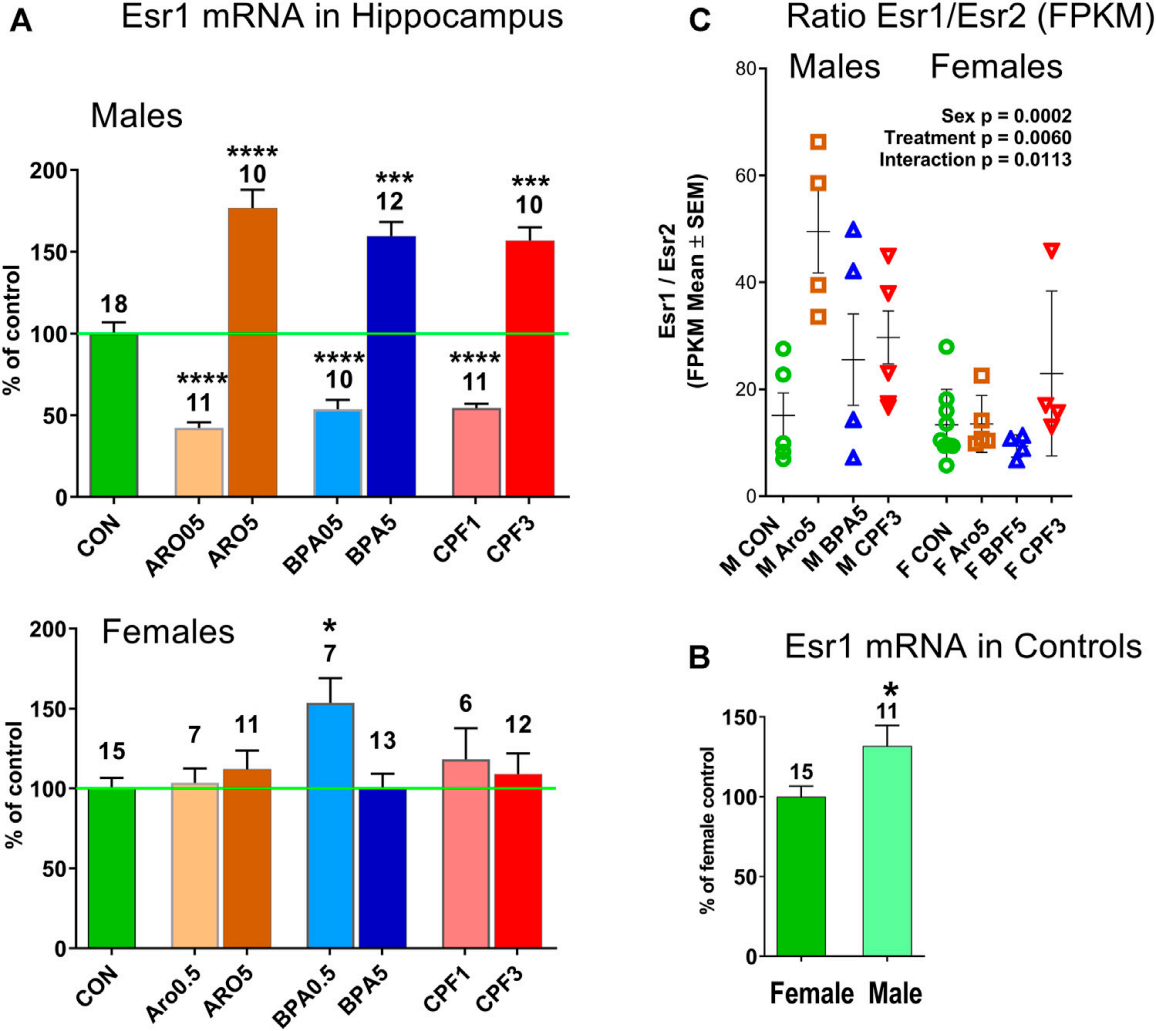
**(A)** Estrogen receptor-alpha (ER-alpha, Esr1) in hippocampus of male and female offspring at postnatal day 6. Quantitative RT PCR, mean ± S.E.M, number of samples. ANOVA, ****p < 0.0001, ***p < 0.001, *p < 0.05. **(B)** Esr1 mRNA level is higher (131.8%) in male than female hippocampus. Quantitative RT PCR, mean ± S.E.M, number of samples. Unpaired *t* test, *p* < 0.05. **(C)** Ratio of ER-alpha (Esr1)/ER-beta (Esr2) in hippocampus of individual male and female offspring is increased in males by treatment with Aro, BPA and CPF at the higher dose level. RNAseq data, FPKM (fragments per kilobase per million reads), two-way ANOVA, treatment p = 0.0060, sex p = 0.0002, interaction treatment x sex p = 0.0113. CON: controls, ARO0.5, ARO5: Aroclor 1254 0.5 mg/kg and 5 mg/kg, BPA0.5, BPA5: bisphenol A 0.5 mg/kg and 5 mg/kg, CPF1, CPF3: chlorpyrifos 1 mg/kg and 3 mg/kg.

The transcriptomics data set indicated increased ER-alpha expression in males but not females after exposure to the higher dose of all three chemicals, and no significant change in the lower dose groups. ER-beta (Esr2) levels remained unchanged (Supplementary Table S8). An analysis of a larger number of hippocampus samples by real time RT PCR confirmed the increased expression of ER-alpha mRNA in male offspring exposed to the chemicals at the higher doses, but revealed a decrease of expression after exposure to the lower doses (Figure 5A). ER-alpha mRNA levels in female hippocampus remained unchanged, except for a significant increase after 0.5 mg/kg BPA. Thus, the two sexes showed opposite responses to BPA. Control levels of ER-alpha mRNA were significantly higher in male hippocampus (131.8% of female level, Figure 5B), in line with previous reports of this developmental phase (Ivanova and Beyer, 2000). The ratio of ER-alpha to ER-beta expression (FPKM values of transcriptomics) differed between sexes (p = 0.0002) (Figure 5C). It was increased in hippocampus of males by all three treatments at the higher dose level (p = 0.0060), but unchanged in females, resulting in a significant sex × treatment interaction (p = 0.0113). ER-alpha/ER-beta ratios of controls were similar in both sexes. No effect was seen in hippocampus of males in the lower dose-range (Supplementary Figure S5).

### Target Genes of Sox6 and Sexually Dimorphic Responses in Interneurons

**FIGURE 6:**
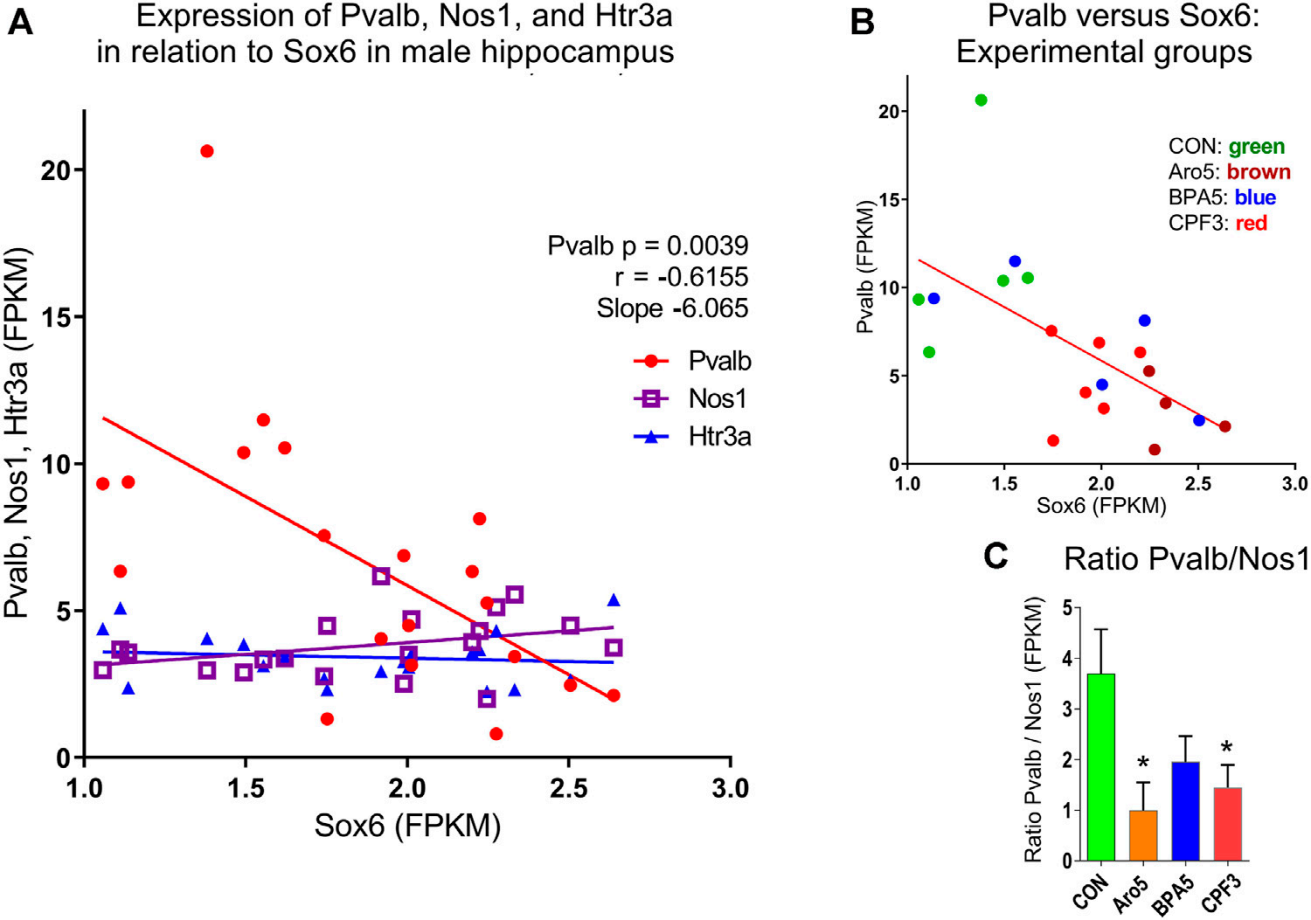
Relationship between Sox6 and interneuron markers expressed in individual hippocampus samples of male offspring at postnatal day 6 (RNAseq data, FPKM values). **(A)** Inverse correlation between expression of Parvalbumin (Pvalb) expressed in medial ganglionic eminence (MGE)-derived interneurons and Sox6, p = 0.0039, r = −0.6155, slope = −6.065. No correlation between Sox6 and levels of neuronal nitric oxide synthase (Nos1) expressed in subgroup of MGE-derived interneurons, or serotonergic ionotropic receptor Htr3a expressed in caudal ganglionic eminence-derived interneurons, both independent of Sox6. **(B)** Correlation between Pvalb and Sox6 with identification of treatment groups. C. Expression ratio of Pvalb/Nos1, mean ± S.E.M, N = 5-6.

The developmental stage studied here corresponds to a period, when Sox6 is involved in differentiation of postmitotic interneuron subtypes, specifically, of medial ganglionic eminence (MGE)-derived parvalbumin (Pvalb)- and somatostatin (Sst)-expressing interneurons (Tricoire et al., 2011; Kelsom and Lu, 2013; Hu et al., 2017). FPKM values of individual hippocampus samples from male offspring of controls and high-dose treatment groups revealed a highly significant inverse correlation between Pvalb and Sox6, whereas no such correlation was seen between individual values of Sox6 and neuronal nitric oxide synthase (Nos1) or between Sox6 and the serotonergic ionotropic receptor Htr3a (Figure 6). The Pvalb/Nos1 expression ratio (FPKM values) was significantly reduced by Aroclor 1254 and chlorpyrifos, and showed a similar tendency with bisphenol A. Nos1 is expressed by some MGE-derived hippocampal interneurons, but is unaffected by conditional removal of Sox6 (Jaglin et al., 2012). Htr3a is a marker of CGE-derived, Sox6-independent interneurons (Vucurovic et al., 2010; Touzot et al., 2016). Transcriptomics analysis indicated a significant down-regulation of Pvalb by the higher doses of Aroclor 1254 and chlorpyrifos (Table 5), while real time RT PCR analysis of a different set of samples showed significantly reduced Pvalb mRNA levels after the higher doses of Aroclor 1254 and bisphenol A (Figure 3). According to real time RT PCR analysis, the lower dose of the three treatments may also have been active, but the effect did not reach statistical significance (Figure 3). Nos1 was slightly but significantly up-regulated by Aroclor 1254 (Table 5), possibly also by chlorpyrifos (Fdr >0.1). Sst expression was not significantly changed (Table 5).

**TABLE 5:** Genes involved in development of different types of GABAergic hippocampal interneurons in male offspring (transcriptomics)^a^.

Gene	Aroclor 1254			Bisphenol A			Chlorpyrifos		
	Log ratio	% CON	*p* Value *Fdr* *^b^*	Log ratio	% CON	*p* Value *Fdr* *^b^*	Log ratio	% CON	*p* Value *Fdr* *^b^*
**Medial ganglionic eminence (MGE)-derived interneuron progenitors** ^**c**^ ^**,**^ ^**d**^									
Sox6-dependent^c^									
Pvalb ^e^	−0.5709	67.32	*7.90E-04 7.02E-03*	−0.2607	83.47	*1.31E-01 4.29E-01*	−0.4823	71.58	*5.45E-03 4.75E-02*
Sst	−0.0659	95.53	*6.50E-01 8.06E-01*	−0.0592	95.98	*6.73E-01 8.73E-01*	−0.0583	96.04	*6.70E-01 8.29E-01*
Sox6-independent									
Nos1 (nNOS)	0.3519	127.62	*2.16E-02 8.64E-02*	0.1840	113.60	*2.17E-01 5.43E-01*	0.2889	122.17	*4.70E-02 1.85E-01*
NPY^f^	0.0596	104.21	*7.33E-01 NA*	0.1033	107.42	*5.55E-01 NA*	−0.2525	83.94	*1.49E-01 NA*
**Caudal ganglionic eminence (CGE)- and preoptic area-derived interneuon progenitors** ^**c**^ ^**,**^ ^**d**^									
Sox6-independent									
Calb2 Calretinin	−0.2357	84.93	*1.70E-01 3.60E-01*	−0.0262	98.20	*8.77E-01 9.59E-01*	−0.2895	81.82	*8.48E-02 2.62E-01*
CCK	0.0131	100.91	*9.36E-01 9.69E-01*	0.1012	107.27	*5.27E-01 7.92E-01*	0.2048	115.25	*1.91E-01 4.22E-01*
Htr3a	−0.0782	94.73	*6.09E-01 7.79E-01*	−0.3677	77.50	*1.37E-02 1.25E-01*	−0.2951	81.50	*4.24E-02 1.74E-01*
VIP	0.0874	106.24	*6.13E-01 7.82E-01*	0.1535	111.23	*3.69E-01 6.84E-01*	0.1751	112.90	*3.01E-01 5.44E-01*
Reln	0.1550	111.34	1.44E-01 *3.23E-01*	0.1390	110.11	1.70E-01 *4.88E-01*	0.1958	114.54	4.48E-02 *1.79E-01*

a Hippocampus of female offspring did not show significant changes in Pvalb, Sst, Nos1, NPY, Calb2, Htr3a, CCK, VIP, or Reln.

Pvalb: parvalbumin, Sst: somatostatin, Nos1; neuronal nitric oxid synthase, NPY: neuropeptide Y, Calb2: calbindin 2, CCK: cholecystokinin, Htr3a: serotonergic ionotropic receptor Htr3a, VIP: vasoactive intestinal peptide, Reln: reelin.

b Fdr: False discovery rate.

c Wonders and Anderson, 2006; Gelman et al., 2009; Vucurovic et al., 2010; Tricoire et al., 2011; Jaglin et al., 2012; Kelsom and Lu, 2013; Touzot et al., 2016; Hu et al., 2017.

d Expression levels of Nkx2-1, Dlx1, Dlx2, Dlx5, Dlx6, Lhx6, Lhx8, Gsx1, Gsx2, and Mash1, involved in development of MGE- and/or CGE-derived interneurons (Kelsom and Lu, 2013), were unchanged.

e Real time RT PCR analysis of Pvalb in Figure 3.

f NPY is expressed in MGE-, CGE-, and POA-derived interneurons (Wonders and Anderson 2006; Gelman et al., 2009; Kelsom and Lu, 2013).

Sox6-independent NPY, expressed in MGE-, CGE-, and preoptic area-derived hippocampal interneurons (Wonders and Anderson, 2006; Gelman et al., 2009; Kelsom and Lu, 2013), as well as markers of CGE-dependent interneurons (Tricoire et al., 2011; Kelsom and Lu, 2013; Touzot et al., 2016), which are Sox6-independent, did not exhibit significant expression changes in male hippocampus (Table 5; *p* < 0.05, Fdr <0.05)). In hippocampus of female offspring, the aforementioned interneuron markers remained unchanged (Supplementary Table S6).

## Discussion

The PCB mixture Aroclor 1254, bisphenol A, and chlorpyrifos, which exhibit endocrine disrupting properties and partly overlap but also differ in their mechanisms of action and effect patterns, all interfere with development of learning and memory, when administered to rodents during pre- and/or neonatal life (Roegge et al., 2000; Levin et al., 2001, 2002; Widholm et al., 2001; Aldridge et al., 2005; Sugawara et al., 2006; Miyagawa et al., 2007; Colciago et al., 2009; Johnson et al., 2009; Xu et al., 2010; Jašarevic et al., 2011; Turgeman et al., 2011; Chen et al., 2012; Kumar and Thakur, 2014; Johnson et al., 2016). We asked the question whether the three chemicals might share common gene expression patterns and signaling pathways that might be linked with development of hippocampus, which is essential for memory functions. Transcriptomics in hippocampus of postnatal day (PND) 6 revealed significant similar effects of the three chemicals on GO processes related to early neurodevelopment, but only in male offspring. Of particular interest are similar effects of all three chemicals on several Sox and Pou genes involved in cell-autonomous control of development, Sox6, Sox11, Pou2f2/Oct2, and Pou3f2/Brn2. This class of genes seems to represent a new focus with respect to neurodevelopmental effects of EDCs. We are only aware of a report by Gauger et al. (2004), who observed upregulation of Oct1 (Pou2f1) in cerebral cortex of rat fetuses exposed to Aroclor 1254. In PND 6 hippocampus, Oct1 was not affected by Aroclor 1254, but was upregulated by bisphenol A and chlorpyrifos. The fact that all three chemicals affected expression of the four genes of the Sox and Pou families, and in the same manner, suggests that the effect may be relevant to alterations in hippocampus development leading to impaired memory functions. There also were GO processes and genes in developing hippocampus exhibiting differential responses to the three chemicals (Supplementary Table S4).

The higher dose of the three chemicals, leading to upregulation of Sox6, Sox11, Pou2f2/Oct2, and Pou3f2/Brn2 mRNAs, corresponds to a level impairing learning and memory in rodent offspring (Roegge et al., 2000; Levin et al., 2001, 2002; Widholm et al., 2001; Aldridge et al., 2005; Sugawara et al., 2006; Miyagawa et al., 2007; Johnson et al., 2009; Xu et al., 2010; Jašarevic et al., 2011; Turgeman et al., 2011; Chen et al., 2012; Kumar and Thakur, 2014; Johnson et al., 2016). Transciptomics analysis of the low-dose groups did not yield significant results, possibly because of high inter-individual variability (false discovery rates above 0.05). Yet, real time RT PCR analysis of Sox6, Sox11, and Pou2f1/Oct1 showed an analogous upregulation of mRNA levels by bisphenol A (but not Aroclor 1254 or chlorpyrifos) also at the lower dose level, which is about 10 times higher than the lowest levels consistently found to affect memory development in rodents (Nakamura et al., 2012; Kumar and Thakur, 2014, see Supplementary Table S1).

Transcriptomics revealed a marked sex difference: None of the aforementioned genes showed an expression change in female hippocampus. In the case of Sox6, a sexually dimorphic effect of the three chemicals on miR-24 may play a role. The notion of a link between ER-alpha, miR-24, and Sox6, indicated by our network analyses, postulating an effect of ER-alpha activation on miR-24 on the one hand, and a suppressive effect of miR-24 on Sox6 expression on the other hand, was based on data from non-neural human cell lines and mouse pancreatic beta cells (Maillot et al., 2009; Melkman-Zehavi et al., 2011; Ferraro et al., 2012; Klinge, 2012). In line with this concept, all three chemicals caused a decrease in miR-24-3p and an increase in Sox6 mRNA level in male hippocampus. The inverse correlation between miR-24-3p and Sox6 mRNA level of individual samples was highly significant (p = 0.0097), suggesting an interaction of miR-24-3p with Sox6 in the same cell. In mammalian cells, mRNA levels reflect the action of microRNAs (Guo et al., 2010). In female hippocampus, both, miR-24-3p and its potential target Sox6 were unchanged, indicating a sex difference in responsiveness of miR-24-3p. miR-24 has recently been reported to exhibit sex-dependent responses to diethylhexylphthalate, with suppression in male and induction in female hippocampus (Luu et al., 2017). Hormone-sensitive microRNAs may represent an important novel target of EDCs in developing brain.

ER-alpha also exhibited sex differences in baseline expression levels and response to treatment. In keeping with earlier data of the same postnatal stage (Ivanova and Beyer, 2000), control ER-alpha mRNA levels were higher in males than females. The increased ER-alpha expression caused by the higher dose level of the three chemicals in males corresponds to the effect of estrogen observed in neonatal rat hippocampal slices of undefined sex (Rune et al., 2002). It resulted in a sex-specific increase of the ER-alpha/ER-beta ratio in males. Provided the expression change was reflected at protein level, this effect might have facilitated estrogenic actions of bisphenol A, PCBs or their metabolites, and chlorpyrifos (Hazarika et al., 2020). Yet, the inverse dose-response exhibited by ER-alpha in males and, in the case of 0.5 mg/kg bisphenol A, also in females, makes a direct link to the changes in downstream genes doubtful. Because of insufficient tissue material, analysis of miR-24-3p was not possible in the low-dose groups. However, neither Sox6, the presumed target of miR-24-3p, nor its downstream target Pvalb, showed an inverse dose-response. Rather, we consider the changes in ER-alpha as parallel events; estrogenic actions would also be possible at baseline ER-alpha levels.

The basis for the general low responsiveness of genes in female hippocampus at PND 6 remains unclear. EDCs are known to influence hippocampal structures in both sexes, but data on the first postnatal week are scarce. Ten out of 11 publications reviewed by Rebuli and Patisaul (2016) and a microarray analysis (Royland et al., 2008) reporting neonatal data, were limited to males or did not distinguish between sexes, and the gene expression study in neonatal females perinatally exposed to bisphenol A included only that sex (Facciolo et al., 2005). At PND 6, certain developmental processes in hippocampus are more advanced in females, as indicated by the higher expression ratio of KCC2/NKCC1, that governs the switch of excitatory to inhibitory action of GABA (Nuñez and McCarthy, 2007; Watanabe and Fukuda, 2015). The three chemicals did not affect the KCC2/NKCC1 ratio, thus, did not appear to change the development stage reached by the females, but the sensitivity of hippocampus to treatment effects may have differed between the developmental stages reached by females and males. In marked contrast to males, gene expression in PND 6 females showed high false discovery rates of the majority of genes, suggesting a combination of high inter-individual variability and small magnitude of gene expression changes. A possible role of developmental stage is indicated by the report of Levin and coworkers (2001, 2002) that memory function was impaired by chlorpyrifos after treatment between postnatal days 1–4 in male rats and after treatment between gestational days 17–20 in females.

The differences in susceptibility of neonatal hippocampus in male and female offspring could also result from specific sex differences in estrogenic mechanisms. Expression of both estrogen receptors is higher in males than females (Ivanova and Beyer, 2000), and analyses of cell proliferation suggest that the hippocampus of male rat neonates may be more sensitive to a weak activation of estrogenic mechanisms (Bowers et al., 2010). The fact that developmental hippocampus is able of *de novo* synthesis of sex steroids (Amateau et al., 2004; Kretz et al., 2004; Nuñez and McCarthy, 2009), and at the same time, may as well be influenced by circulating steroid hormones, complicates an interpretation of EDC effects. At peripubertal stage, there again are prominent effects of prenatal exposure to EDCs in male rat offspring, such as changes in expression and DNA methylation of BDNF (brain-derived neurotrophic factor) after prenatal bisphenol A exposure (Kundakovic et al., 2015), but gene expression changes have also been observed in female hippocampus at that stage, specifically, genes related to serotonin turnover, after the same treatment (Matsuda et al., 2013). Adult female hippocampus shows distinct, estradiol-dependent, gene expression changes during the estrous cycle (Iqbal et al., 2020), illustrating estrogen-sensitivity of hippocampal genes in the female at later life stages.

Investigations of memory functions in adult rodent offspring of both sexes perinatally exposed to one of the three chemicals show a heterogeneous picture with effects only in males or females, or differential or similar effects in males and females (Supplementary Table S1). In children, two studies reported a link between prenatal bisphenol A exposure and preferential impairment of cognitive functions in boys (Braun et al., 2017; Bornehag et al., 2021). A higher susceptibility of boys has also been observed with respect to other types of behaviors in relation to prenatal phthalate exposure (Engel et al., 2010). Many investigations do not report differences between boys and girls (e.g., Boucher et al., 2009, PCBs). One reason for the difficulty to associate hormone-regulated effects in developing hippocampus with behavioral outcome in adulthood could be that the behavioral parameters are not sufficiently detailed. Sex-dependent features of adult hippocampal function result from the activity of hormone-sensitive genes at multiple developmental stages, and, in addition, are influenced by circulating as well as locally synthetized sex steroids in a stage-dependent manner. This cannot be understood along a scheme of sexual brain differentiation leading to either male-or female-type behaviours, but rather, may lead to a mosaic of multiple functional processes in hippocampus, as well as in cortical and subcortical regions interacting with hippocampus, that are more or less influenced by sex steroids during development.

The change in Sox6 expression in male hippocampus was accompanied by an expression change of the Sox6-regulated Pvalb gene. Sox6 exerts different functions depending on developmental stage. It maintains stemness of neural precursor/progenitor cells (Ohta et al., 2013) and oligodendrocyte progenitors (Baroti et al., 2016), and, at later stages, plays a role in specification of cortical and hippocampal interneurons (Batista-Brito et al., 2009; Tricoire et al., 2011; Kelsom and Lu, 2013). Specifically, Sox6 regulates subtype determination MGE-derived postmitotic interneurons by suppressing differentiation of parvalbumin expressing interneurons and inducing differentiation of somatostatin expressing interneurons (Hu et al., 2017). The highly significant (p = 0.0039) inverse correlation between Sox6 and Pvalb indicates that the increased expression of Sox6 was functionally effective to downregulate Pvalb expression at the level of individual hippocampus samples. No relationship was seen between levels of Sox6 and of Nos1 (nNos), which is expressed by MGE-derived interneurons but is Sox6-independent in hippocampus (Jaglin et al., 2012), and between Sox6 and Htr3a, a marker of CGE-derived interneurons (Vucurovic et al., 2010). Developmental exposure to the three chemicals thus appeared to elicit a cascade of events involving a specific interneuron subtype in male but not female hippocampus, with downregulation of miR-24, upregulation of Sox6, and downregulation of Pvalb. There were no expression changes of markers of CGE-derived interneurons, or genes expressed by MGE-derived interneurons but not regulated by Sox6. An imbalance between interneurons is indicated by an increased Pvalb/Nos1 expression ratio. In the absence of morphological data, we do not know whether this represents a change in the number of Pvalb expressing cells, or in intracellular level of Pvalb mRNA. Since it had not been possible to perform perfusion-fixation, we tried to do immunocytochemistry on cryostat sections of brains frozen on dry-ice, but because of the ice crystals formed during freezing, tissue quality turned out to be insufficient for morphometric analysis. If the number of Pvalb interneurons was affected, this might disturb the balance between Pvalb interneurons (fast spiking basket cells and axo-axonic chandelier cells) (Donato et al., 2013), and other types of interneurons.

Our investigation revealed yet another target mechanism, by which hippocampal interneuron development may be disturbed: Expression of Nrg1 and its receptor Erbb4, which control interneuron migration (Faux et al., 2012), was altered, Nrg1 by Aroclor 1254 and bisphenol A, Erbb4 by the PCB mixture and chlorpyrifos. Thus, hippocampal interneuron development emerges as a potentially important target of EDCs.

## Conclusion and Regulatory Perspectives

Our investigation disclosed a potential role of genes of the Sox and Pou families involved in cell-autonomous regulation of development, which so far seem to have received little attention in neurodevelopmental effects of EDCs. These genes were found to be indirect but common targets of three different EDCs known to impair hippocampal functions in adult offspring, suggesting that they occupy a central position in the pathogenetic process leading to adverse behavioral outcome. At the developmental stage studied, these genes exhibited a marked sexual dimorphism. In the case of Sox6, the sex difference was correlated with sex-dependent regulation of miR-24. This underscores the potential relevance of microRNAs in developmental effects of EDCs. Developing interneurons were found to represent an important EDC target, as demonstrated by the inverse correlation between mRNA expression levels of Sox6 and of parvalbumin, the marker of a Sox6-regulated subpopulation of hippocampal interneurons. A role of interneurons in developmental effects of EDCs is further suggested by expression changes of Nrg1 and its receptor Erbb4, regulating interneuron migration. It is evident that our descriptive findings need to be further corroborated by mechanistic studies analyzing the proposed EDC–miR–target gene interactions at the cellular level, and by morphological analyses of interneuron populations.

There is a great need to better link *in vitro* models designed for regulatory purposes, with adverse outcomes *in vivo*. Omics data could represent such links, but for that purpose, we need more information on the relationship between effects of EDCs on molecular processes during early stages of brain development, and delayed adverse outcome, because *in vitro* models focus on early developmental processes. Information on omics obtained at later postnatal and adult stages is of interest for the interpretation of functional alterations, but such analyses miss effects on early possible targets such as morphogenetic genes, which are visible only during restricted time-windows. Transcriptomics represents only one part of the approach. Since gene expression patterns change continuously throughout ontogeny, transcriptomics analyses provide information on signaling pathways targeted by a chemical at a given developmental stage, which can then be compared to *in vitro* systems, and they disclose potential target genes that could be studied by epigenetics. It is the information obtained with such a combined approach, complemented by metabolomics, which should increase the predictive value of *in vitro* and *in vivo* models.

## Preliminary Reports

Preliminary reports were presented at Copenhagen Workshop on Endocrine Disrupters (COW 2017), Copenhagen, 2–5 May 2017, EUROTOX 2017, Bratislava, 10–13 September 2017, and 10th International Meeting on Steroids and Nervous System, Torino, Italy, 16–20 February 2019.

## Data Availability

The RNAseq data have been uploaded to GEO/ENA, ID: GSE180054.
