# Supplementary material for: Converging Effects of Three Different Endocrine Disrupters on Sox and Pou Gene Expression in Developing Rat Hippocampus: Possible Role of microRNA in Sex Differences
Source: Front Genet. 2021 Nov 11;12:718796. doi: 10.3389/fgene.2021.718796 (PMC8632217; doi:10.3389/fgene.2021.718796)
Supplement: Supplementary file 1 [file DataSheet1.PDF]

Supplemental Table 1. Literature data used for dose selection

Compound	Species Route	Treatment period	Doses studied mg/kg	Effective doses mg/kg	Sex studied	Sex affected	Effects on learning/memory or anxiety	Reference
PCB Mixture: Aroclor 1254								
Aroclor 1254	Mouse oral	GD6 - PND20 every 3 days	6, 18, 54	18	M	M	Morris water maze	Sugawara et al., 2006
Aroclor 1254	Rat oral	GD6 - PND21	6	6	MF	M	Radial arm maze	Roegge et al., 2000
Aroclor 1254	Rat oral	GD6 - PND21	6	6	MF	MF differentially affected	Reversal learning	Widholm et al., 2001
Aroclor 1254	Rat oral	GD6 - PND21	6	6	M	M	Long-term potentiation positive. Morris water maze negative	Gilbert et al., 2000
PCB52, 138, 180	Rat oral	GD7 - PND21	1	1	MF	MF	Y maze	Boix et al., 2010
PCB126 + 138 + 153 + 180	Rat s.c.	GD15 – PND21	10	10	MF	M	Passive avoidance learning positive. Morris water maze negative	Colciago et al., 2009
Aroclor 1254	Rat oral	GD6 - PND21	6	Ineffective	MF	No M+F data combined	Operant conditioning negative	Taylor et al., 2002
Bisphenol A								
Bisphenol A	Mouse oral	Mating – PND21	160, 0.0024	160, 0.0024	M	M	Passive avoidance retention (No dose-response). Anxiety negative	Miyagawa et al. 2007
Bisphenol A	Mouse oral	2 wk before mating – PND21	8	8	MF	M	Barnes spatial maze	Jasarevic et al. 2011
Bisphenol A	Mouse oral	GD7 – PND21	0.5, 5, 50	5	M	M	Morris water maze	Xu et al. 2010
Bisphenol A	Mouse oral	GD7-20 as well as PND1-14	0.4, 4	4	MF	MF	Anxiety (elevated plus maze)	Xu et al. 2012

Bisphenol A	Rat oral	GD6-GD22 + pups PND1 – PND21	0.0025, 0.025, 2.5	2.5	MF	MF differentially affected	Barnes maze	Johnson et al. 2016
Bisphenol A	Rat oral drinkin g water	GD0 – PND21	1.5	1.5	MF	MF	Loss of sex difference in open field time and in passive avoidance behavior	Kubo et al., 2001
Bisphenol A	Mouse oral	GD3 – GD21	0.2	0.2	F	F	Anxiety (elevated plus maze). Radial-arm maze and Barnes maze negative	Ryan et al. 2006
Bisphenol A	Mouse oral	GD7 – PND21	0.05	0.05	M	M	Morris water maze	Kumar et al., 2014
Bisphenol A	Mouse s.c.	GD10 – PND20	0.0002 5	0.00025	MF	M	Anxiety (Open field)	Matsuda et al., 2012
Bisphenol A	Mouse s.c.	GD0 – PND21	0.02	ineffective	MF	No	Morris water maze negative. Anxiety negative	Nakamura et al., 2012
Bisphenol A	Rat oral	GD6–GD21 + pups PND1 – PND21	0.025, 0.0025	ineffective	MF	No	Morris water maze negative, Barnes spatial maze negative	Ferguson et al. 2012
Bisphenol A	Rat oral drinkin g water	GD0 – PND21	0.015	ineffective	MF	No	Passive avoidance negative. Anxiety negative	Fujimoto et al. 2006
Chlorpyrifos								
Chlorpyrifos	Mouse s.c.	GD9 – GD18	3	3	MF	MF	Morris water maze	Turgeman et al. 2011
Chlorpyrifos	Rat s.c.	GD17 - GD20	1, 5	1, 5 (1 ≅ 5)	MF	F	Radial maze	Levin et al. 2002
Chlorpyrifos	Rat s.c.	PND1 – PND4	1, 5	1, 5 (1 ≅ 5)	MF	M	Radial maze	Levin et al. 2001
Chlorpyrifos	Rat s.c.	PND1 – PND4	1	1	MF	M	Radial maze	Aldridge et al. 2005
Chlorpyrifos	Mouse s.c.	GD17 – GD20	0.1, 5	0.1, 5	MF	F	Foraging maze positive. Radial maze negative	Haviland et al. 2010
Chlorpyrifos	Mouse s.c.	GD13 – GD 17	1, 5	5	MF	M	T maze lose-shift errors	Chen et al., 2012

Chlorpyrifos	Mouse oral	GD15 – PND14	0.2, 1, 5	1, 5	F	F	Anxiety	Braquenier et al. 2010
Chlorpyrifos	Rat oral	GD6 – PND10	0.3, 5	ineffective	MF	No M+F data combined	T maze	Maurissen et al. 2000

References

Aldridge et al. Environ. Health Perspect. 113:527–531 (2005)
Boix et al. Neurosci. 167 : 994 –1003 (2010)
Braquenier et al. Neurotoxicol. Teratol. 32 : 234–239 (2010)
Chen et al., Brain Res. 1474 : 19-28 (2013)
Colciago et al. Toxicol. Appl. Pharmacol. 239 : 46–54 (2009)
Ferguson et al. Neurotoxicol. Teratol. 34: 598-606 (2012)
Fujimoto et al. Brain Res. 1068 : 49-55 (2006)
Gilbert et al. Toxicol. Sci. 57 : 102-111 (2000)
Haviland et al. Reprod. Toxicol. 29 : 74–79 (2010)
Jasarevic et al. PNAS 108 : 11715–11720 (2011)
Johnson et al. Horm. Behav. 80 : 139-148 (2016)
Kubo et al. Neurosci. Lett. 304 : 73-76 (2001)
Kumar et al. PLoS ONE 9(10): e110482. doi:10.1371/journal.pone.0110482
Levin et al. Dev. Brain Res. 130 : 83–89 (2001)
Levin et al. Neurotoxicol. Teratol. 24 : 733-741 (2002)
Matsuda et al. Progr. Neuro-Psychopharmacol. Biol. Psychiat. 39 : 273-279 (2012)
Maurissen et al. Toxicol. Sci. 57 : 250-263 (2000)
Miyagawa et al. Neurosci. Lett. 418 : 236–241 (2007)
Nakamura et al. Brain and Devel. 34 : 57-63 (2012)
Roegge et al. Toxicol. Sci. 57 : 121-130 (2000)
Ryan et al. Horm. Behav. 50 : 85–93 (2006)
Sugawara et al. Arch. Toxicol. 80: 286–292 (2006)
Taylor et al. Neurotoxicol. Teratol. 24 : 511-518 (2002)
Turgeman et al. J. Neurosci. Res. 89:1185–1193 (2011)
Widholm et al. Toxicol. Appl. Pharmacol. 174 : 188–198 (2001)
Xu et al. Horm. Behav. 58 : 326–333 (2010)
Xu et al. Horm. Behav. 62 : 480-490 (2012)

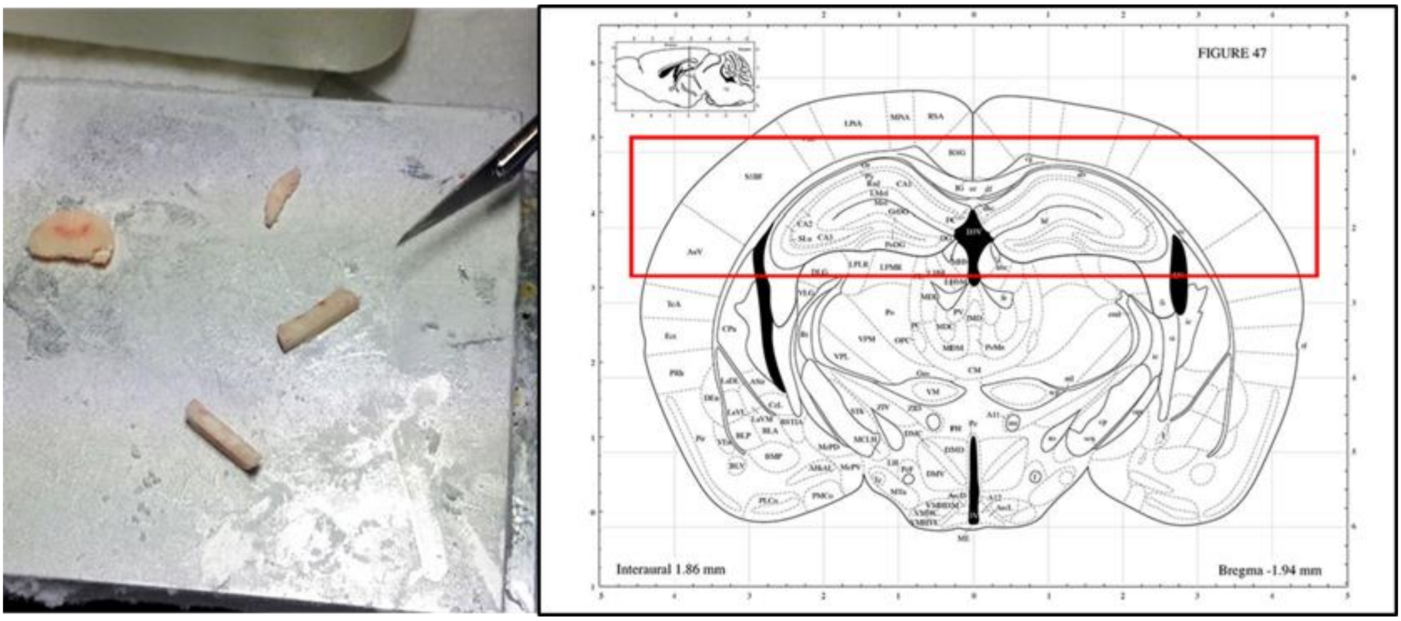

Supplemental Fig. 1. Left side: Frontal 1.0 mm thick slice of rat brain at postnatal day 6, cut at a level immediately posterior to the optic chiasm in a stainless steel rat brain matrix (Zivic Instruments) cooled to below –10°C by dry ice. Rectangular tissue piece comprising hippocampus plus laterally adjacent cortical tissue (Right side: red square) dissected under microscopic control on stainless steel plate cooled by Pelletier element (–10°C).

Supplemental Table 2. RNA-seq: Adapter sequences
Oligonucleotide sequences for TruSeq™ RNA and DNA Sample Prep Kits
TruSeq Universal Adapter
5’ AATGATACGGCGACCACCGAGATCTACACTCTTTCCCTACACGACGCTCTTCCGATCT
TruSeq™ Adapters
TruSeq Adapter, Index 1
5’ GATCGGAAGAGCACACGTCTGAACTCCAGTCACATCACGATCTCGTATGCCGTCTTCTGCTTG
TruSeq Adapter, Index 2
5’ GATCGGAAGAGCACACGTCTGAACTCCAGTCACCGATGTATCTCGTATGCCGTCTTCTGCTTG
TruSeq Adapter, Index 3
5’ GATCGGAAGAGCACACGTCTGAACTCCAGTCACTTAGGCATCTCGTATGCCGTCTTCTGCTTG
TruSeq Adapter, Index 4
5’ GATCGGAAGAGCACACGTCTGAACTCCAGTCACTGACCAATCTCGTATGCCGTCTTCTGCTTG
TruSeq Adapter, Index 5
5’ GATCGGAAGAGCACACGTCTGAACTCCAGTCACACAGTGATCTCGTATGCCGTCTTCTGCTTG
TruSeq Adapter, Index 6
5’ GATCGGAAGAGCACACGTCTGAACTCCAGTCACGCCAATATCTCGTATGCCGTCTTCTGCTTG
TruSeq Adapter, Index 7
5’ GATCGGAAGAGCACACGTCTGAACTCCAGTCACCAGATCATCTCGTATGCCGTCTTCTGCTTG
TruSeq Adapter, Index 8
5’ GATCGGAAGAGCACACGTCTGAACTCCAGTCACACTTGAATCTCGTATGCCGTCTTCTGCTTG
TruSeq Adapter, Index 9
5’ GATCGGAAGAGCACACGTCTGAACTCCAGTCACGATCAGATCTCGTATGCCGTCTTCTGCTTG
TruSeq Adapter, Index 10
5’ GATCGGAAGAGCACACGTCTGAACTCCAGTCACTAGCTTATCTCGTATGCCGTCTTCTGCTTG
TruSeq Adapter, Index 11
5’ GATCGGAAGAGCACACGTCTGAACTCCAGTCACGGCTACATCTCGTATGCCGTCTTCTGCTTG
TruSeq Adapter, Index 12
5’ GATCGGAAGAGCACACGTCTGAACTCCAGTCACCTTGTAATCTCGTATGCCGTCTTCTGCTTG
TruSeq Adapter, Index 13
5’GATCGGAAGAGCACACGTCTGAACTCCAGTCACAGTCAACAATCTCGTATGCCGTCTTCTGCTTG
TruSeq Adapter, Index 14
5’ GATCGGAAGAGCACACGTCTGAACTCCAGTCACAGTTCCGTATCTCGTATGCCGTCTTCTGCTTG
TruSeq Adapter, Index 15
5’ GATCGGAAGAGCACACGTCTGAACTCCAGTCACATGTCAGAATCTCGTATGCCGTCTTCTGCTTG
TruSeq Adapter, Index 16
5’ GATCGGAAGAGCACACGTCTGA ACTCCAGTCACCCGTCCCGATCTCGTATGCCGTCTTCTGCTTG
TruSeq Adapter, Index 18 4
5’ GATCGGAAGAGCACACGTCTGAACTCCAGTCACGTCCGCACATCTCGTATGCCGTCTTCTGCTTG
TruSeq Adapter, Index 19
5’ GATCGGAAGAGCACACGTCTGAACTCCAGTCACGTGAAACGATCTCGTATGCCGTCTTCTGCTTG
TruSeq Adapter, Index 20
5’ GATCGGAAGAGCACACGTCTGAACTCCAGTCACGTGGCCTTATCTCGTATGCCGTCTTCTGCTTG
TruSeq Adapter, Index 21
5’ GATCGGAAGAGCACACGTCTGAACTCCAGTCACGTTTCGGAATCTCGTATGCCGTCTTCTGCTTG
TruSeq Adapter, Index 22
5’ GATCGGAAGAGCACACGTCTGAACTCCAGTCACCGTACGTAATCTCGTATGCCGTCTTCTGCTTG
TruSeq Adapter, Index 23
5’ GATCGGAAGAGCACACGTCTGAACTCCAGTCACGAGTGGATATCTCGTATGCCGTCTTCTGCTTG
TruSeq Adapter, Index 25
5’ GATCGGAAGAGCACACGTCTGAACTCCAGTCACACTGATATATCTCGTATGCCGTCTTCTGCTTG
TruSeq Adapter, Index 27
5’ GATCGGAAGAGCACACGTCTGAACTCCAGTCACATTCCTTTATCTCGTATGCCGTCTTCTGCTTG

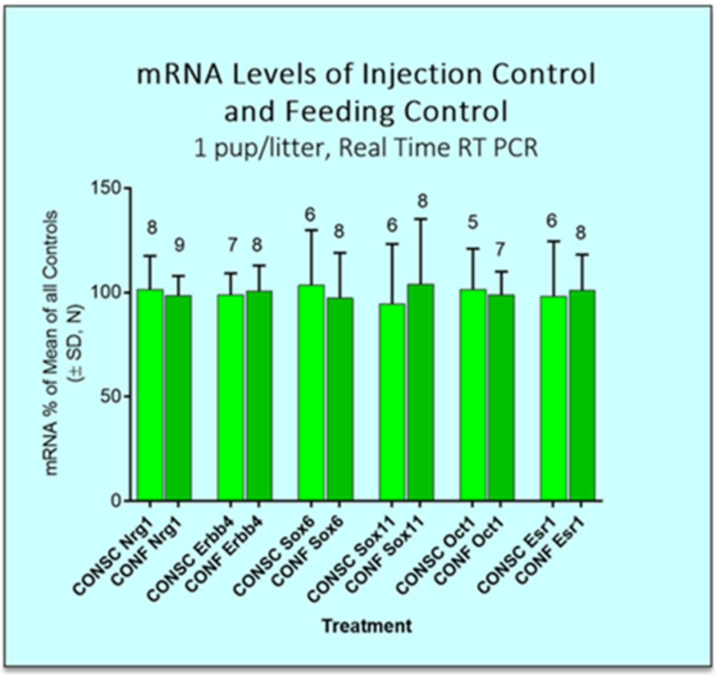

Supplemental Fig. 2. mRNA expression levels of 6 target genes (Nrg1, Erbb4, Sox6, Sox11, Oct1, Esr1) determined by Real Time RT PCR in injection controls (CONSC) and feeding controls (CONF). Mean ± SD of 5-9 samples taken from different litters, and number of litters.

Supplemental Table 3. Sequences of primers and TaqMan probes used for real time RT PCR, with NCBI-Accession Numbers¨		
Parvalbumin (Pvalb)<br>NM_022499	FP<br>RP<br>Probe	5'-GCT TCA TTG AGG AGG ATG AG-3'<br>5'-CCA GAG TGG AGA ATT CTT CAA C-3'<br>5'-TCC TTG TCT CCA GCA GCC A-3'
Estrogen receptor 1 (Esr1)<br>NM_012689	FP<br>RP<br>Probe	5'-GGC TTT GGG GAC TTG AAT C-3'<br>5'-CCT TGA TTC CTG TCC AAG AG-3'<br>5'-TCA CCT TCT GGA GTG TGC CT-3'
SRY-box transcription factor 11 (Sox11)<br>NM_053349	FP<br>RP<br>Probe	5'-ACC TCA TGT TCG ACC TGA-3'<br>5'-GCT GAA GGA ATC CAG GTC-3'<br>5'-CAC CAG CGA CAG GGA CAG GTT CC-3'
SRY-box transcription factor 6 (Sox6)<br>NM_001024751	FP<br>RP<br>Probe	5'-GCA AGA ACA GAT CGC AAG AC-3'<br>5'-CCC TGA ACC TGC TGG ATC-3'<br>5'-CAG CAG CAA CCT TCT ACA GCA GCA GCA C-3'
Erb-b2 receptor tyrosine kinase 4 (Erbb4)<br>NM_021687	FP<br>RP<br>Probe	5'- CAA CGG CTG AGA TGT TTG ATG A -3'<br>5'- ACC TCT GAG TGC TAC TGT CCT CTT G -3'<br>5'- CCT GCT GTA ATG GTA CCC TGC GAA AGC -3'
Neuregulin 1(Nrg1)<br>NM_001271130	FP<br>RP<br>Probe	5'-GTG GTC GGC ATC ATG TGT GT-3'<br>5'-CGA AGC CGA TCA TGA AGC TT-3'<br>5'-TAC TGC AAA ACC AAG AAG CAG CGG CA-3'
POU class 2 homeobox 1 (Pou2f1)<br>NM_001100639	FP<br>RP<br>Probe	5'-GCT CAA TCT TTA AAT GTA CAG TCT A-3'<br>5'-CAG TGT GAG CCC AGT TAT C-3'<br>5'-AGA TTC GCA GCA GTC AAG CCA GC-3'

Supplemental Table 4. Enrichment by gene ontology processes in hippocampus of male rat offspring: Significant difference from control group (p < 0.01, threshold 0.5; MetaCore)

			**Aroclor 1254** 5 mg/kg			**Bisphenol A** 5 mg/kg			**Chlorpyrifos** 3 mg/kg		
# [1)]	GO Process	Total	p-value	FDR	In Data	p-value	FDR	In Data	p-value	FDR	In Data
1	**nervous system development**	3138	1.397E-22	8.558E-19	144	2.751E-08	7.777E-05	41	5.758E-14	2.608E-10	79
2	**behavior**	906	6.124E-21	1.875E-17	68	3.744E-03	4.947E-02	12	1.164E-08	1.759E-06	31
3	**cell-cell signaling**	1518	1.282E-20	2.617E-17	90	5.135E-04	1.834E-02	19	3.936E-09	8.103E-07	43
4	**regulation of signaling**	4074	1.049E-19	1.607E-16	163	2.671E-02	1.287E-01	31	2.736E-07	1.652E-05	76
5	**regulation of cell communication**	4125	1.439E-19	1.763E-16	164	3.120E-02	1.392E-01	31	2.129E-07	1.418E-05	77
6	**synaptic signaling**	651	2.426E-19	2.476E-16	55	2.533E-02	1.267E-01	8	3.791E-08	4.033E-06	25
7	**single-organism behavior**	703	3.514E-19	3.075E-16	57	1.428E-02	9.560E-02	9	2.450E-09	7.171E-07	28
8	**brain development**	1133	1.562E-17	1.196E-14	71	1.296E-04	1.019E-02	17	1.621E-08	1.998E-06	35
9	**cell development**	2198	3.179E-17	2.163E-14	105	3.836E-05	5.818E-03	27	1.204E-08	1.759E-06	53
10	**head development**	1187	5.107E-17	3.128E-14	72	2.252E-04	1.221E-02	17	5.133E-08	4.843E-06	35
11	**central nervous system development**	1453	5.756E-17	3.205E-14	81	1.022E-04	1.004E-02	20	1.076E-08	1.740E-06	41
12	**neurogenesis**	2202	1.033E-16	4.550E-14	104	1.567E-06	1.188E-03	30	2.179E-12	3.289E-09	61
13	**system development**	5761	1.048E-16	4.550E-14	197	1.274E-05	4.003E-03	53	4.236E-08	4.170E-06	100
14	**generation of neurons**	2072	1.136E-16	4.550E-14	100	1.428E-06	1.188E-03	29	5.322E-13	1.205E-09	60
15	**chemical synaptic transmission**	627	1.263E-16	4.550E-14	50	2.081E-02	1.129E-01	8	3.076E-07	1.741E-05	23
16	**trans-synaptic signaling**	627	1.263E-16	4.550E-14	50	2.081E-02	1.129E-01	8	3.076E-07	1.741E-05	23
17	**anterograde trans-synaptic signaling**	627	1.263E-16	4.550E-14	50	2.081E-02	1.129E-01	8	3.076E-07	1.741E-05	23
18	**cognition**	452	1.939E-16	6.597E-14	42	1.161E-02	8.481E-02	7	9.917E-08	7.744E-06	20
19	**locomotory behavior**	312	2.216E-16	7.144E-14	35	5.164E-05	6.636E-03	9	1.261E-09	5.191E-07	19
20	**anatomical structure morphogenesis**	2968	2.710E-16	8.299E-14	125	1.957E-04	1.177E-02	31	4.395E-10	2.488E-07	68
21	**regulation of membrane potential**	524	3.025E-16	8.822E-14	45	2.401E-02	1.225E-01	7	5.581E-08	5.066E-06	22
22	**central nervous system neuron differentiation**	286	7.119E-16	1.982E-13	33	9.527E-04	2.282E-02	7	2.929E-10	1.895E-07	19
23	**regulation of signal transduction**	3603	1.836E-15	4.890E-13	140	1.622E-02	9.893E-02	29	1.021E-06	4.301E-05	68
24	**adult behavior**	263	3.037E-15	7.750E-13	31	4.175E-01	5.536E-01	2	5.420E-10	2.727E-07	18
25	**regulation of ion transmembrane transport**	586	4.091E-15	1.002E-12	46	2.121E-01	3.701E-01	5	1.694E-03	1.272E-02	15
26	**forebrain development**	587	4.358E-15	1.010E-12	46	4.683E-03	5.493E-02	9	3.927E-07	2.145E-05	22
27	**learning or memory**	406	4.450E-15	1.010E-12	38	2.325E-02	1.200E-01	6	4.298E-07	2.290E-05	18
28	**neuron differentiation**	1397	7.321E-15	1.579E-12	75	5.209E-04	1.834E-02	18	9.100E-11	6.869E-08	44

29	**regulation of transmembrane transport**	620	7.514E-15	1.579E-12	47	2.457E-01	3.988E-01	5	2.898E-03	1.917E-02	15
30	**cell differentiation**	4654	7.733E-15	1.579E-12	165	2.562E-05	4.527E-03	45	4.230E-08	4.170E-06	86
31	**negative regulation of biological process**	6196	1.340E-14	2.648E-12	201	4.454E-04	1.773E-02	51	1.199E-08	1.759E-06	107
32	**multicellular organism development**	6564	1.831E-14	3.399E-12	209	1.291E-04	1.019E-02	55	3.042E-07	1.741E-05	107
33	**regulation of localization**	3406	2.117E-14	3.814E-12	132	4.119E-02	1.602E-01	26	4.620E-05	8.867E-04	60
34	**glutamate receptor signaling pathway**	59	3.002E-14	5.253E-12	16	4.070E-02	1.600E-01	2	3.662E-03	2.209E-02	4
35	**regulation of transport**	2576	3.158E-14	5.373E-12	109	2.013E-02	1.129E-01	22	1.934E-06	7.027E-05	53
36	**regulation of synaptic transmission, glutamatergic**	93	3.265E-14	5.405E-12	19	9.051E-02	2.389E-01	2	6.938E-07	3.307E-05	9
37	**rhythmic process**	524	3.380E-14	5.449E-12	42	3.156E-01	4.577E-01	4	5.611E-02	1.487E-01	10
38	**regulation of ion transport**	883	3.576E-14	5.593E-12	56	2.025E-01	3.629E-01	7	2.880E-05	6.102E-04	24
39	**regulation of synaptic transmission, GABAergic**	50	3.652E-14	5.593E-12	15	2.379E-01	3.921E-01	1	1.506E-05	3.589E-04	6
40	**developmental process**	7509	4.068E-14	6.078E-12	229	2.014E-04	1.186E-02	60	8.094E-08	6.546E-06	120
41	**modulation of synaptic transmission**	484	5.243E-14	7.646E-12	40	4.887E-01	6.151E-01	3	5.170E-06	1.561E-04	18
42	**organ morphogenesis**	1335	6.848E-14	9.755E-12	71	8.588E-04	2.130E-02	17	3.214E-08	3.550E-06	38
43	**positive regulation of nucleobase-containing compound metabolic process**	2329	8.089E-14	1.126E-11	101	1.332E-02	9.188E-02	21	9.625E-04	8.304E-03	41
44	**regulation of biological quality**	4752	1.134E-13	1.543E-11	164	6.875E-02	2.066E-01	33	7.815E-04	7.025E-03	72
45	**single-organism developmental process**	7411	1.535E-13	2.044E-11	225	5.262E-04	1.834E-02	58	1.485E-07	1.102E-05	118
46	**cellular developmental process**	4905	1.958E-13	2.552E-11	167	4.390E-05	5.909E-03	46	2.289E-07	1.503E-05	87
47	**negative regulation of cellular process**	5777	3.250E-13	4.073E-11	187	2.998E-04	1.367E-02	49	2.308E-08	2.680E-06	101
48	**positive regulation of nitrogen compound metabolic process**	2493	3.646E-13	4.466E-11	104	1.426E-02	9.560E-02	22	3.408E-03	2.094E-02	41
49	**regulation of hormone secretion**	444	3.766E-13	4.522E-11	37	9.374E-02	2.409E-01	5	1.496E-08	1.998E-06	21
50	**positive regulation of nucleic acid-templated transcription**	1941	4.247E-13	4.908E-11	88	3.797E-03	4.947E-02	20	1.572E-03	1.213E-02	35
51	**positive regulation of transcription, DNA-templated**	1941	4.247E-13	4.908E-11	88	3.797E-03	4.947E-02	20	1.572E-03	1.213E-02	35
52	**positive regulation of cellular process**	6317	4.473E-13	5.074E-11	199	7.648E-03	7.031E-02	47	4.716E-05	9.013E-04	96
53	**negative regulation of cell communication**	1743	4.613E-13	5.137E-11	82	1.278E-02	9.010E-02	17	1.056E-04	1.649E-03	36
54	**animal organ development**	4380	4.770E-13	5.218E-11	153	6.714E-04	1.954E-02	39	4.804E-06	1.460E-04	76
55	**regulation of multicellular organismal development**	2544	5.316E-13	5.712E-11	105	1.006E-03	2.327E-02	26	5.968E-09	1.040E-06	59
56	**negative regulation of signaling**	1723	6.857E-13	7.241E-11	81	1.150E-02	8.426E-02	17	1.833E-04	2.457E-03	35
57	**anatomical structure development**	7102	7.088E-13	7.358E-11	216	3.047E-04	1.367E-02	57	7.560E-07	3.494E-05	112

58	**regulation of multicellular organismal process**	3767	7.724E-13	7.885E-11	137	5.093E-03	5.731E-02	32	1.652E-09	6.234E-07	78
59	**positive regulation of RNA biosynthetic process**	1963	7.938E-13	7.970E-11	88	4.309E-03	5.304E-02	20	1.901E-03	1.397E-02	35
60	**ion transmembrane transport**	1071	8.758E-13	8.535E-11	60	5.248E-01	6.448E-01	6	2.679E-03	1.808E-02	22
61	**regulation of phospholipase C activity**	72	8.851E-13	8.535E-11	16	3.239E-01	4.648E-01	1	4.866E-09	9.582E-07	10
62	**single-multicellular organism process**	7480	8.918E-13	8.535E-11	224	1.274E-03	2.572E-02	57	1.855E-06	6.830E-05	115
63	**localization**	6331	1.136E-12	1.070E-10	198	1.055E-01	2.555E-01	41	2.556E-04	3.054E-03	93
64	**neuromuscular process**	159	1.333E-12	1.219E-10	22	5.542E-02	1.856E-01	3	3.226E-04	3.708E-03	8
65	**positive regulation of cellular biosynthetic process**	2475	1.390E-12	1.252E-10	102	6.717E-03	6.481E-02	23	5.166E-03	2.792E-02	40
66	**regulation of phospholipase activity**	116	2.147E-12	1.906E-10	19	4.681E-01	5.969E-01	1	4.910E-07	2.498E-05	10
67	**synapse maturation**	15	2.361E-12	2.066E-10	9	2.914E-03	4.184E-02	2	8.765E-11	6.869E-08	7
68	**positive regulation of molecular function**	2438	3.272E-12	2.822E-10	100	5.611E-03	5.823E-02	23	3.601E-04	4.007E-03	44
69	**regulation of intracellular signal transduction**	2265	3.769E-12	3.188E-10	95	9.904E-03	8.075E-02	21	1.268E-06	4.994E-05	49
70	**positive regulation of RNA metabolic process**	2020	3.799E-12	3.188E-10	88	5.909E-03	5.924E-02	20	3.042E-03	1.935E-02	35
71	**positive regulation of biosynthetic process**	2520	4.130E-12	3.373E-10	102	8.301E-03	7.379E-02	23	4.119E-03	2.389E-02	41
72	**telencephalon development**	394	6.442E-12	5.192E-10	33	5.687E-03	5.823E-02	7	5.864E-06	1.735E-04	16
73	**intracellular signal transduction**	2096	1.117E-11	8.884E-10	89	1.612E-01	3.194E-01	15	3.058E-03	1.935E-02	36
74	**tissue morphogenesis**	873	1.171E-11	9.198E-10	51	7.839E-03	7.093E-02	11	6.787E-08	5.597E-06	29
75	**regulation of peptide hormone secretion**	358	1.220E-11	9.460E-10	31	1.298E-01	2.821E-01	4	6.672E-08	5.597E-06	18
76	**regulation of synapse structure or activity**	382	1.352E-11	1.035E-09	32	5.688E-02	1.874E-01	5	8.613E-07	3.833E-05	17
77	**learning**	256	1.693E-11	1.280E-09	26	5.062E-02	1.780E-01	4	2.178E-05	4.907E-04	12
78	**cellular response to endogenous stimulus**	1770	1.873E-11	1.399E-09	79	2.498E-01	4.031E-01	12	2.410E-02	8.506E-02	28
79	**regulation of peptide secretion**	366	2.150E-11	1.587E-09	31	1.375E-01	2.903E-01	4	9.297E-08	7.387E-06	18
80	**positive regulation of signaling**	2089	2.286E-11	1.667E-09	88	5.811E-02	1.874E-01	17	4.236E-06	1.331E-04	45
81	**regulation of nervous system development**	1193	2.548E-11	1.836E-09	61	2.220E-05	4.483E-03	19	3.155E-11	3.573E-08	41
82	**developmental maturation**	326	2.975E-11	2.119E-09	29	2.014E-03	3.369E-02	7	2.563E-06	8.726E-05	15
83	**tube development**	931	3.777E-11	2.659E-09	52	1.549E-04	1.176E-02	15	5.919E-09	1.040E-06	32
84	**positive regulation of macromolecule biosynthetic process**	2257	4.290E-11	2.974E-09	92	4.635E-03	5.493E-02	22	5.734E-03	3.013E-02	37
85	**response to endogenous stimulus**	2549	4.369E-11	2.974E-09	100	4.066E-01	5.440E-01	15	2.165E-02	7.799E-02	38
86	**regulation of secretion by cell**	1026	4.661E-11	3.138E-09	55	5.186E-02	1.780E-01	10	5.751E-08	5.066E-06	32
87	**positive regulation of cell communication**	2119	4.828E-11	3.214E-09	88	6.465E-02	2.008E-01	17	6.138E-06	1.782E-04	45
88	**regulation of insulin secretion**	311	4.957E-11	3.265E-09	28	8.885E-02	2.357E-01	4	4.701E-08	4.530E-06	17

89	**positive regulation of biological process**	6811	5.563E-11	3.625E-09	203	3.036E-02	1.392E-01	47	5.525E-04	5.500E-03	97
90	**regulation of response to stimulus**	5073	6.172E-11	3.979E-09	163	1.373E-01	2.901E-01	33	4.857E-05	9.243E-04	81
91	**regulation of hormone levels**	774	7.328E-11	4.627E-09	46	1.272E-01	2.780E-01	7	1.053E-05	2.725E-04	23
92	**single organism signaling**	7067	8.553E-11	5.346E-09	208	2.604E-01	4.106E-01	42	1.409E-02	5.907E-02	92
93	**signaling**	7069	8.795E-11	5.428E-09	208	2.611E-01	4.112E-01	42	1.419E-02	5.936E-02	92
94	**cell surface receptor signaling pathway involved in cell-cell signaling**	640	8.863E-11	5.428E-09	41	7.219E-04	2.001E-02	11	6.413E-05	1.135E-03	19
95	**transmembrane transport**	1427	9.817E-11	5.954E-09	67	3.676E-01	5.094E-01	9	2.881E-03	1.913E-02	27
96	**response to organic cyclic compound**	1764	1.018E-10	6.110E-09	77	2.858E-02	1.342E-01	16	1.185E-03	9.848E-03	33
97	**cell communication**	7219	1.065E-10	6.331E-09	211	3.156E-01	4.577E-01	42	8.922E-03	4.258E-02	95
98	**regulation of developmental process**	3283	1.260E-10	7.419E-09	118	2.766E-02	1.310E-01	26	7.871E-07	3.601E-05	64
99	**negative regulation of response to stimulus**	2017	1.283E-10	7.446E-09	84	4.433E-02	1.655E-01	17	1.671E-06	6.359E-05	45
100	**regulation of molecular function**	3798	1.289E-10	7.446E-09	131	5.768E-03	5.824E-02	32	1.320E-05	3.164E-04	67

[1)] Rank order corresponds to Aroclor 1254. Bisphenol A and chlorpyrifos show different rank orders.
FDR: False discovery rate.

Supplemental Fig. 3. Enrichment by gene ontology processes in hippocampus of male rat offspring: Similarity of processes ($p < 0.01$, threshold 0.5; MetaCore). Orange (top): Aroclor 1254 (5 mg/kg), blue (center): bisphenol A (5 mg/kg), red (bottom): chlorpyrifos (3 mg/kg)

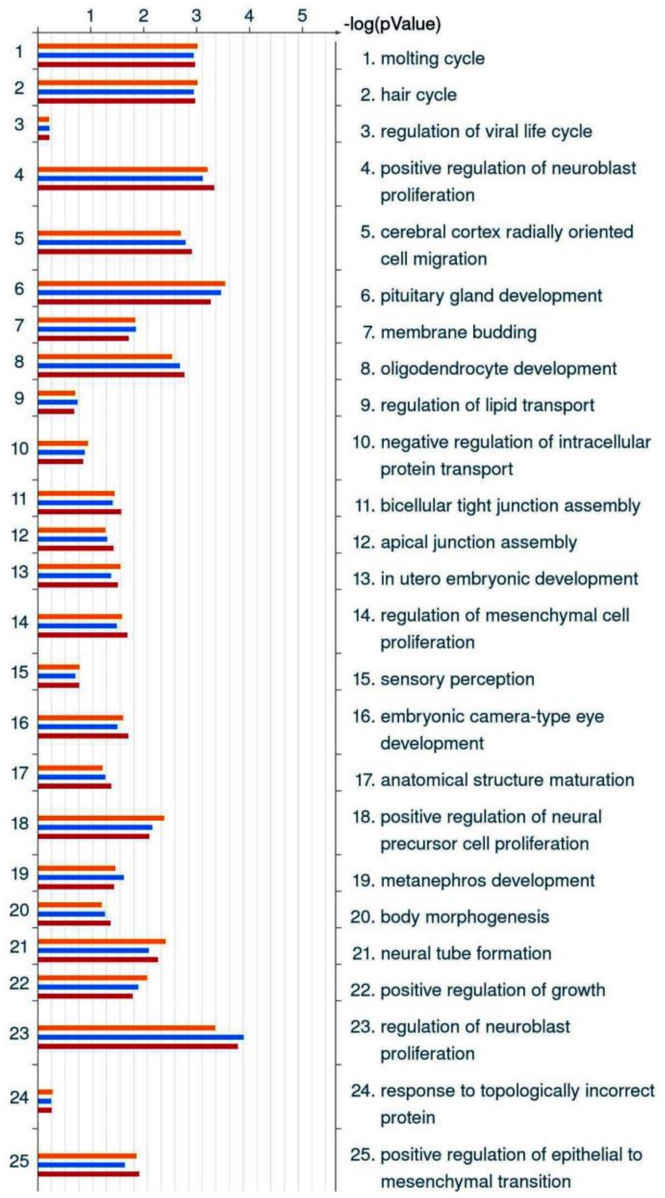

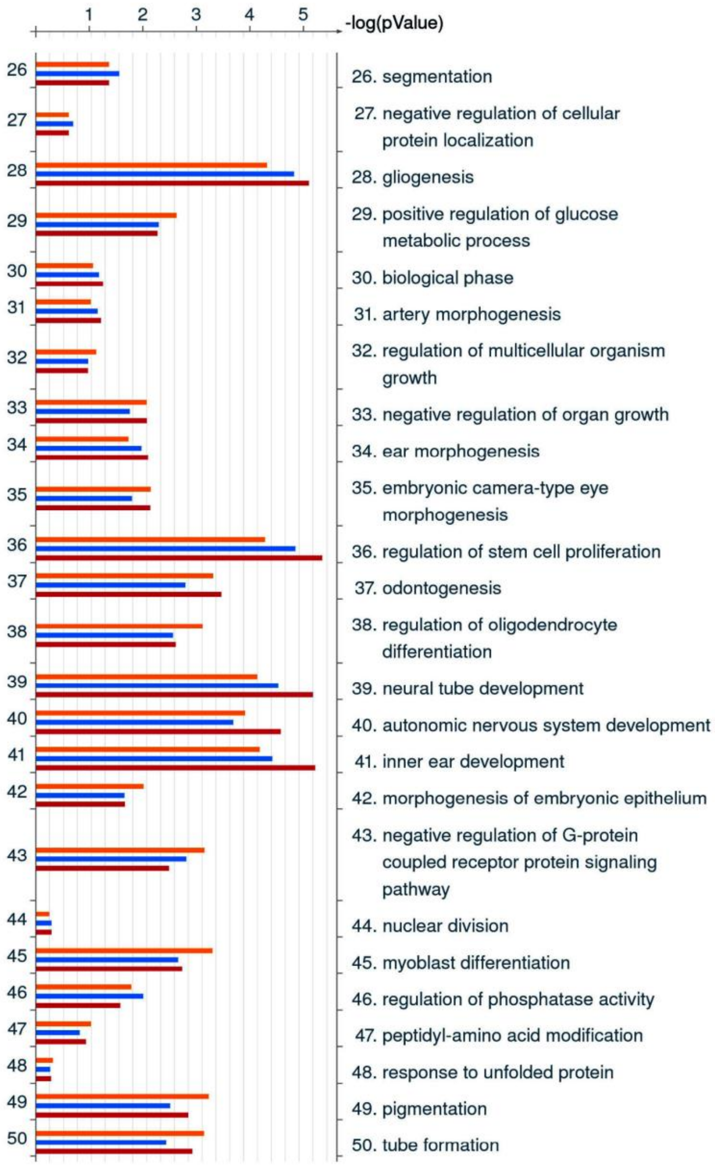

Supplemental Fig. 3 (continued). Enrichment by gene ontology processes in hippocampus of male rat offspring: Similarity of processes

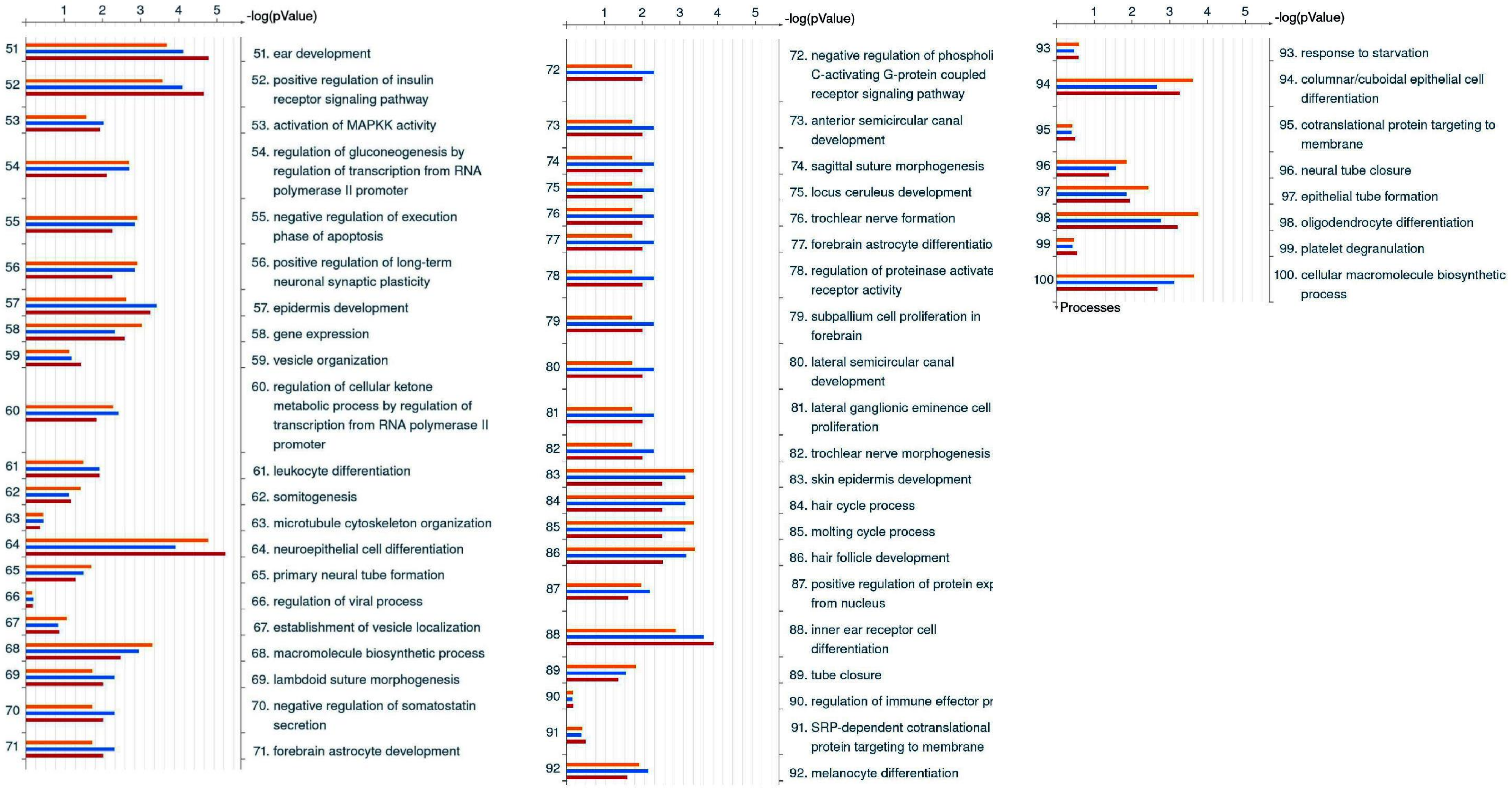

Supplemental Table 5. Main similarly affected gene ontology processes with target genes in hippocampus of male rat offspring (p = 0.01, threshold 0.5)

Regulation of stem cell proliferation

–log(pV) = 4.5 to 5

Gene Symbol	Aro5		BPA5		CPF3	
	Log Ratio	p-value	Log Ratio	p-value	Log Ratio	p-value
Bmpr1a	0.682	4.37E-07	0.501	7.22E-05		
Ago3	1.101	3.39E-13	0.877	1.12E-09	0.736	6.47E-07
Fzd3	1.007	4.97E-13	0.615	3.09E-06	0.589	1.45E-05
Gli3	0.990	1.01E-11	0.667	1.51E-06	0.654	4.29E-06
Chrm2	0.623	1.61E-04				
Irs1	0.913	1.00E-08	0.736	1.37E-06	0.635	4.61E-05
Nfia	0.729	1.70E-06	0.686	2.08E-06	0.596	5.80E-05
Nf1	0.771	1.20E-08	0.581	4.52E-06	0.594	5.40E-06
Notch2	0.702	1.02E-06	0.444	1.41E-03		
Prrx1	0.597	8.46E-06				
Sox11	0.683	7.72E-05	0.368	3.15E-02		

Similarity by process, Thr 0.5 (Log Ratio), p value 0.01, GO no. 36, Suppl. Fig. 3

Neuroepithelial cell differentiation

–log(pV) = 4.5 to 5

Gene Symbol	Aro5		BPA5		CPF3	
	Log Ratio	p-value	Log Ratio	p-value	Log Ratio	p-value
Gli3	0.990	1.01E-11	0.654	4.29E-06	0.667	1.51E-06
Oxtr	-0.594	1.88E-04				
Alg10	1.157	2.16E-11	0.716	2.97E-05	0.919	5.94E-08
Kcnma1	0.664	2.78E-06				
Myo5a	0.816	3.46E-07	0.504	1.27E-03	0.685	7.68E-06
Notch2	0.702	1.02E-06			0.583	1.64E-05
Slc4a7	0.595	1.69E-07			0.624	3.03E-09
Sox11	0.683	7.72E-05			0.525	1.94E-03
Sec24a	1.200	1.28E-12	0.876	1.51E-07	0.952	6.73E-09
Tcf7l2	0.810	2.87E-06	0.642	1.89E-04	0.747	1.13E-05

Similarity by process, Thr 0.5 (Log Ratio), p value 0.01, GO no. 64, Suppl. Fig. 3

Regulation of neuroblast proliferation

–log(pV) = 4

Gene Symbol	Aro5		BPA5		CPF3	
	Log Ratio	p-value	Log Ratio	p-value	Log Ratio	p-value
Fzd3	1.007	4.97E-13	0.589	1.45E-05	0.615	3.09E-06
Gli3	0.990	1.01E-11	0.654	4.29E-06	0.667	1.51E-06
Chrm2	0.623	1.61E-04				
Nf1	0.771	1.20E-08	0.594	5.40E-06	0.581	4.52E-06
Notch2	0.702	1.02E-06			0.583	1.64E-05

Similarity by process, Thr 0.5 (Log Ratio), p value 0.01, GO no. 23, Suppl. Fig. 3

Positive regulation of neuroblast proliferation

–log(pV) = 3.5

Gene Symbol	Aro5		BPA5		CPF3	
	Log Ratio	p-value	Log Ratio	p-value	Log Ratio	p-value
Fzd3	1.007	4.97E-13	0.589	1.45E-05	0.615	3.09E-06
Gli3	0.990	1.01E-11	0.654	4.29E-06	0.667	1.51E-06
Chrm2	0.623	1.61E-04				
Notch2	0.702	1.02E-06			0.583	1.64E-05

Similarity by process, Thr 0.5 (Log Ratio), p value 0.01, GO no. 4, Suppl. Fig. 3

Positive regulation of neural precursor cell proliferation

–log(pV) = 2.5 to 3

Gene Symbol	Aro5		BPA5		CPF3	
	Log Ratio	p-value	Log Ratio	p-value	Log Ratio	p-value
Fzd3	1.007	4.97E-13	0.589	1.45E-05	0.615	3.09E-06
Gli3	0.990	1.01E-11	0.654	4.29E-06	0.667	1.51E-06
Chrm2	0.623	1.61E-04				
Oxtr	-0.594	1.88E-04				
Notch2	0.702	1.02E-06			0.583	1.64E-05

Similarity by process, Thr 0.5 (Log Ratio), p value 0.01, GO no. 18, Suppl. Fig. 3

Cerebral cortex radially oriented cell migration

–log(pV) = 3

Gene Symbol	Aro5		BPA5		CPF3	
	Log Ratio	p-value	Log Ratio	p-value	Log Ratio	p-value
Cdk5r1	0.529	1.01E-04				
Dab1	0.508	4.25E-04	0.580	1.88E-05		
Gli3	0.990	1.01E-11	0.667	1.51E-06	0.654	4.29E-06
Pou3f2	0.648	6.79E-05	0.564	3.07E-04	0.584	2.45E-04

Similarity by process, Thr 0.5 (Log Ratio), p value 0.01, GO no. 5, Suppl. Fig. 3

Gliogenesis

–log(pV) = 4.5 to 5

Gene Symbol	Aro5		BPA5		CPF3	
	Log Ratio	p-value	Log Ratio	p-value	Log Ratio	p-value
Cdk5r1	0.529	1.01E-04				
Dab1	0.508	4.25E-04	0.580	1.88E-05		
Epha4	0.585	3.51E-06	0.554	2.33E-06		
Gli3	0.990	1.01E-11	0.667	1.51E-06	0.654	4.29E-06
Kcnq3	0.930	7.22E-08	0.731	1.58E-05	0.571	8.49E-04
Nfia	0.729	1.70E-06	0.686	2.08E-06	0.596	5.80E-05
Nrg1	0.528	4.91E-05				
Nf1	0.771	1.20E-08	0.581	4.52E-06	0.594	5.40E-06
Notch2	0.702	1.02E-06	0.444	1.41E-03		
Pou3f2	0.648	6.79E-05	0.564	3.07E-04	0.584	2.45E-04
Pten	0.647	3.39E-08				
Sox11	0.683	7.72E-05	0.525	1.94E-03		
Sox6	0.610	1.89E-06				
Tcf7l2	0.810	2.87E-06	0.642	1.89E-04	0.747	1.13E-05

Similarity by process, Thr 0.5 (Log Ratio), p value 0.01, GO no. 28, Suppl. Fig. 3

Oligodendrocyte development

–log(pV) = 3

Gene Symbol	Aro5		BPA5		CPF3	
	Log Ratio	p-value	Log Ratio	p-value	Log Ratio	p-value
Kcnq3	0.930	7.22E-08	0.571	8.49E-04	0.731	1.58E-05
Pten	0.647	3.39E-08				
Sox11	0.683	7.72E-05			0.525	1.94E-03
Tcf7l2	0.810	2.87E-06	0.642	1.89E-04	0.747	1.13E-05

Similarity by process, Thr 0.5 (Log Ratio), p value 0.01, GO no. 8, Suppl. Fig. 3

Supplemental Fig. 4. Enrichment by gene ontology processes in hippocampus of female rat offspring: Similarity of processes (p < 0.01, threshold 0.1; MetaCore). Orange (top): Aroclor 1254 (5 mg/kg), blue (center): bisphenol A (5 mg/kg), red (bottom): chlorpyrifos (3 mg/kg)

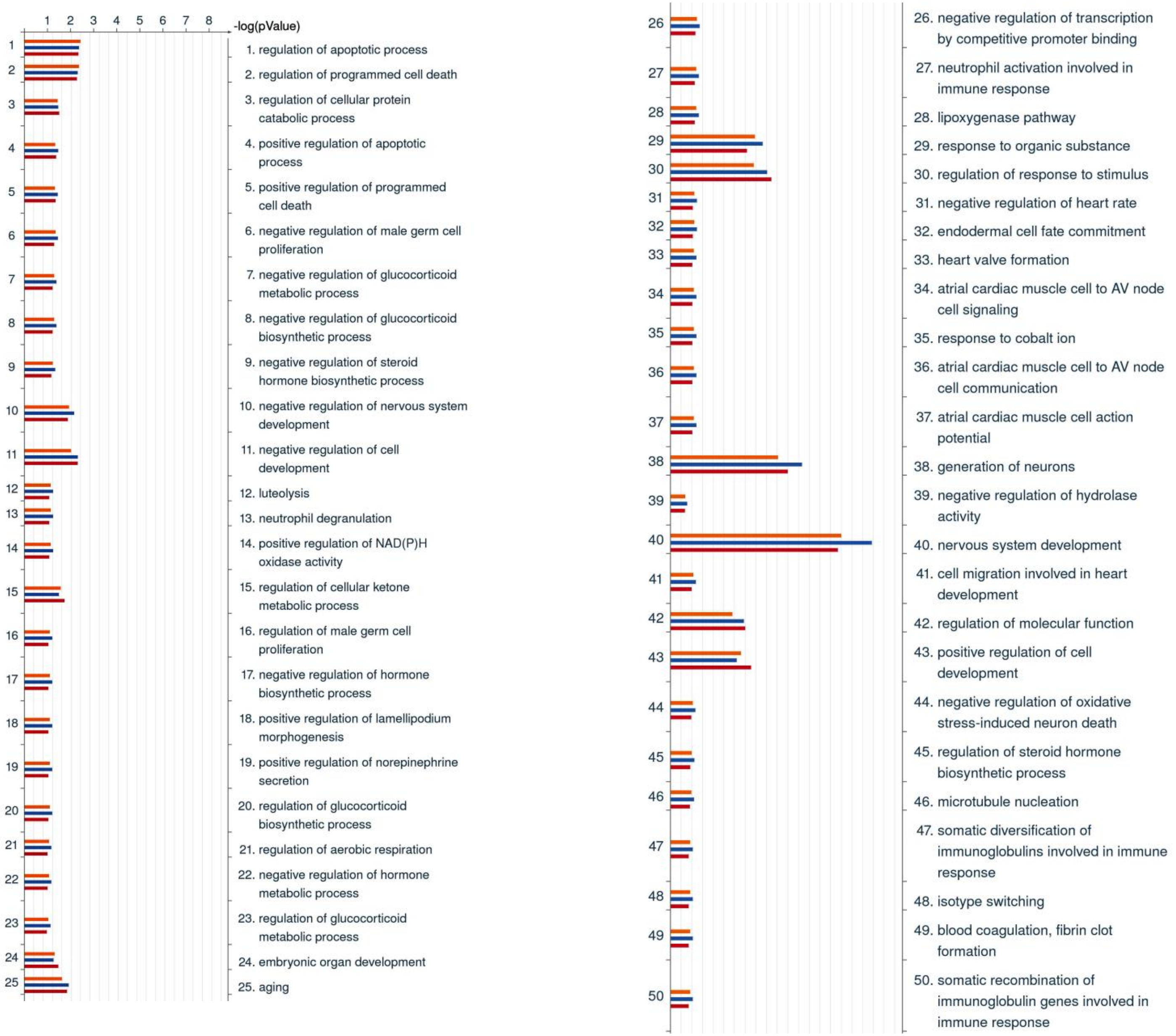

Supplemental Fig. 4 (continued). Enrichment by gene ontology processes in hippocampus of female rat offspring: Similarity of processes

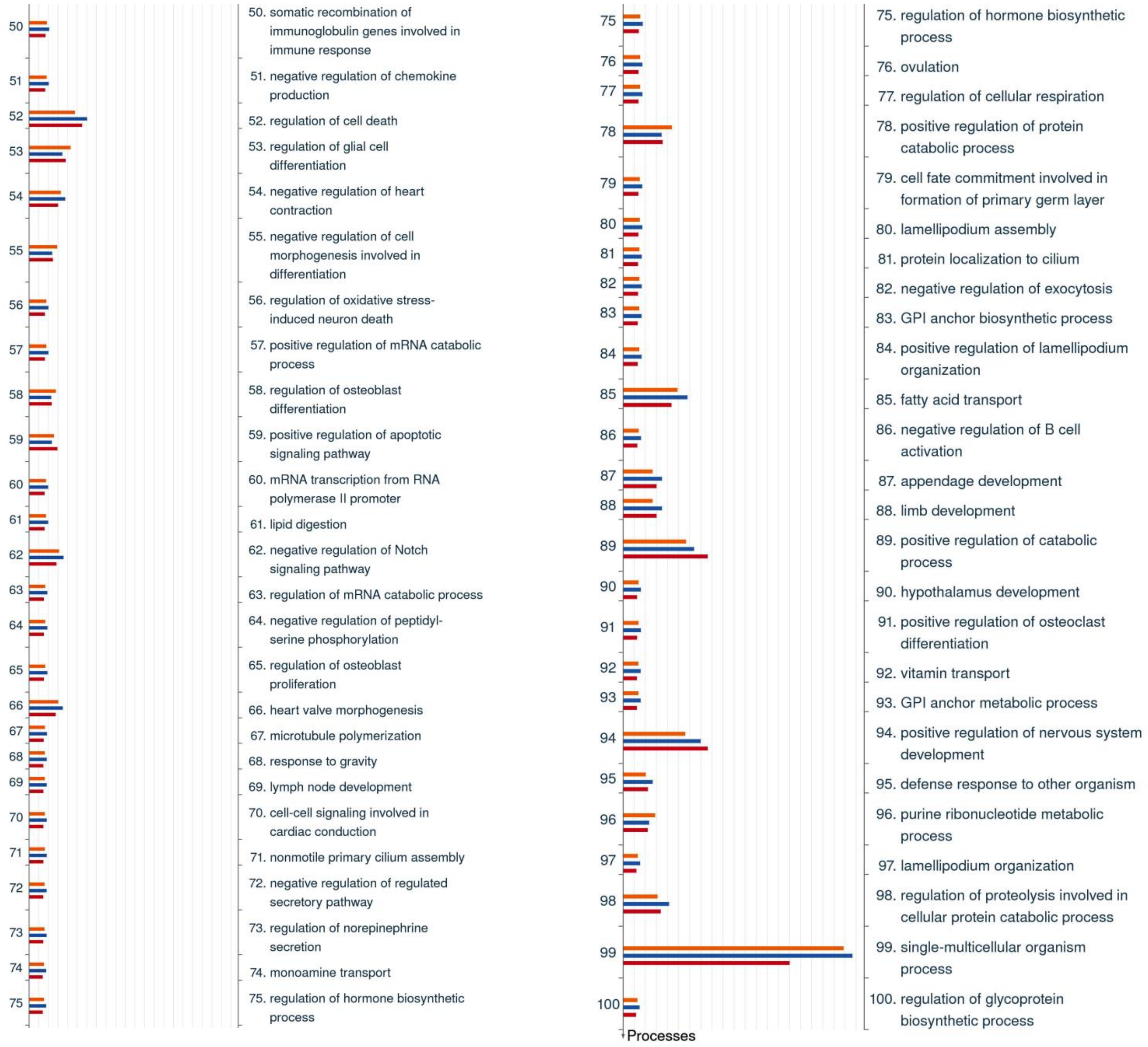

Supplemental Table 6. Hippocampus of female offspring. Expression of genes that were similarly affected by all three treatments in male hippocampus

Gene	Aroclor 1254			Bisphenol A			Chlorpyrifos		
	Log ratio	% CON	p Value<br>*Fdr*[1)]	Log ratio	% CON	p Value<br>*Fdr*[1)]	Log ratio	% CON	p Value<br>*Fdr*[1)]
Genes involved in cell-autonomic regulation of development									
Gli3	–0.0716	95.16	3.98E-01<br>*8.93E-01*	-0.0172	98.82	8.46E-01<br>*9.94E-01*	0.1492	110.90	1.71E-01<br>*7.54E-01*
Pou2f1<br>Oct1	–0.1458	90.39	9.03E-02<br>*7.78E-01*	0.0150	101.04	8.70E-01<br>*9.95E-01*	0.2032	115.12	4.96E-02<br>*6.50E-01*
Pou2f2<br>Oct2	–0.0099	99.31	9.03E-01<br>*9.88E-01*	0.1199	108.67	1.80E-01<br>*9.46E-01*	0.0823	105.87	4.07E-01<br>*8.64E-01*
Pou3f2<br>Brn2	–0.0262	98.20	7.39E-01<br>*9.62E-01*	-0.0200	98.63	8.12E-01<br>*9.92E-01*	–0.0164	98.87	8.74E-01<br>*9.75E-01*
Pou3f3<br>Brn1	–0.0480	96.72	5.58E-01<br>*9.29E-01*	-0.0605	95.90	5.10E-01<br>*9.79E-01*	0.0792	105.64	4.48E-01<br>*8.74E-01*
Sox2	0.1012	107.27	1.62E-01<br>*8.25E-01*	0.1364	109.92	7.56E-02<br>*9.21E-01*	0.1471	110.73	1.01E-01<br>*7.22E-01*
Sox4	0.0024	100.16	9.74E-01<br>*9.96E-01*	0.0249	101.74	7.24E-01<br>*9.92E-01*	0.3083	123.82	1.60E-04<br>*3.56E-01*
Sox5	0.0237	101.66	7.04E-01<br>*9.56E-01*	-0.0909	93.89	1.66E-01<br>*9.45E-01*	0.1196	108.64	1.54E-01<br>*7.47E-01*
Sox6	–0.0880	94.08	3.04E-01<br>*8.60E-01*	0.0563	103.98	5.47E-01<br>*9.80E-01*	0.0133	100.93	9.01E-01<br>*9.82E-01*
Sox11	–0.0406	97.23	6.35E-01<br>*9.42E-01*	0.1826	113.49	4.78E-02<br>*9.07E-01*	–0.1357	91.02	2.30E-01<br>*8.64E-01*
Sox17	0.0594	104.20	4.56E-01<br>*9.07E-01*	0.1438	110.48	9.56E-02<br>*9.41E-01*	–0.0713	95.18	4.69E-01<br>*8.79E-01*
Genes encoding for growth factors and receptors of transcellular signaling pathways									
Nrg1	–0.0066	99.54	9.36E-01<br>*9.93E-01*	0.1253	109.07	1.55E-01<br>*9.45E-01*	0.0595	104.21	5.52E-01<br>*9.05E-01*
Erbb4	–0.1815	88.18	2.98E-02<br>*7.18E-01*	–0.0246	98.31	7.62E-01<br>*9.92E-01*	–0.0159	98.91	8.85E-01<br>*9.79E-01*
Efnb2	–0.05458	96.29	5.01E-01<br>*9.14E-01*	0.0182	101.27	8.36E-01<br>*9.93E-01*	–0.0087	99.40	9.37E-01<br>*9.87E-01*
Eph4	–0.1702	88.87	1.95E-02<br>*6.82E-01*	–0.1423	90.61	6.98E-02<br>*9.20E-01*	–0.0684	95.37	4.29E-01<br>*8.69E-01*
Fzd3	–0.1383	90.86	1.03E-01<br>*7.88E-01*	0.0324	102.27	7.22E-01<br>*9.92E-01*	–0.0364	97.51	7.41E-01<br>*9.53E-01*
Tcf7L2	–0.1651	89.19	4.26E-02<br>*7.26E-01*	–0.0207	98.58	8.07E-01<br>*9.92E-01*	–0.0543	96.31	6.16E-01<br>*9.24E-01*
Notch2	–0.1085	92.76	2.02E-01<br>*8.37E-01*	–0.0002	99.99	9.85E-01<br>*NA*	–0.0007	99.95	9.60E-01<br>*NA*
Esr1	0.0593	104.20	4.81E-01<br>*9.10E-01*	0.0940	106.73	3.05E-01<br>*9.74E-01*	-0.05617	96.18	6.03E-01<br>*9.22E-01*
Esr2	0.0072	100.50	9.00E-01<br>*NA*	0.0870	106.22	1.54E-01<br>*NA*	-0.08843	94.05	2.42E-01<br>*NA*
Sox6-dependent genes expressed by medial ganglionic eminence-derived interneurons									
Pvalb	0.0422	102.97	5.54E-01<br>*9.28E-01*	0.0490	103.45	5.13E-01<br>*9.79E-01*	-0.0357	97.56	6.81E-01<br>*9.39E-01*
Sst	0.0340	102.38	5.89E-01<br>*9.33E-01*	0.1503	110.98	1.26E-02<br>*8.14E-01*	0.1313	109.53	5.09E-02<br>*6.50E-01*

[1)] Fdr: False discovery rate. Efnb2: Ephrin B2, Eph4: ephrin type-A receptor 4

Supplemental Table 7. Magnitude of expression change detected in male hippocampus by RNAseq and qRT PCR

mRNA species	Aroclor 1254 5 mg/kg		Bisphenol A 5 mg/kg		Chlorpyrifos 3 mg/kg	
	RNAseq % of Control	qRT PCR [1)] % of Control	RNAseq % of Control	qRT PCR [1)] % of Control	RNAseq % of Control	qRT PCR [1)] % of Control
Sox6	152.6	167.8	134.2	141.7	135.9	143.8
Sox11	160.5	142.6	129.1	148.3	143.9	160.1
Pou2f1/Oct1	127.5	132.0	126.4	138.0	146.4	134.4
Nrg1	144.1	127.4	139.1	119.0	132.3	117.2
Erbb4	192.2	133.6	136.6	122.1	165.6	143.1
Pvalb	67.3	56.4	83.5	59.9	71.6	68.4

[1)] qRT PCR was performed on hippocampus samples not used for RNAseq.

Supplemental Table 8. Estrogen receptor expression in male and female hippocampus

Gene	Aroclor 1254 5 mg/kg			Bisphenol A 5 mg/kg			Chlorpyrifos 3 mg/kg		
Males									
	Log ratio	% CON	p Value *Fdr2)*	Log ratio	% CON	p Value *Fdr2)*	Log ratio	% CON	p Value *Fdr2)*
Esr1	0.5958	151.13	1.83E-05 *3.67E-04*	0.3096	123.94	2.24E-02 *1.68E-01*	0.4753	139.02	2.85E-04 *6.19E-03*
Esr2	0.0554	103.92	7.48E-01 *NA*	0.2085	115.55	2.22E-01 *NA*	-0.2662	83.15	1.20E-01 *NA*
	Aroclor 1254 0.5 mg/kg			Bisphenol A 0.5 mg/kg			Chlorpyrifos 1 mg/kg		
Esr1	–0.2557	83.76	5.13E-02 *2.49E-01*	–0.0344	97.65	7.94E-01 *9.83E-01*	–0.1067	92.87	4.19E-01 *9.38E-01*
Esr2	0.0054	100.37	9.62E-01 *NA*	0.0767	105.46	4.74E-01 *NA*	0.0112	100.78	9.16E-01 *NA*
Females									
	Aroclor 1254 5 mg/kg			Bisphenol A 5 mg/kg			Chlorpyrifos 3 mg/kg		
Esr1	0.0593	104.20	4.81E-01 *9.10E-01*	0.0940	106.73	3.05E-01 *9.74E-01*	-0.05617	96.18	6.03E-01 *9.22E-01*
Esr2	0.0072	100.50	9.00E-01 *NA*	0.0870	106.22	1.54E-01 *NA*	-0.08843	94.05	2.42E-01 *NA*

No transcriptomics analysis was performed with low-dose groups in females

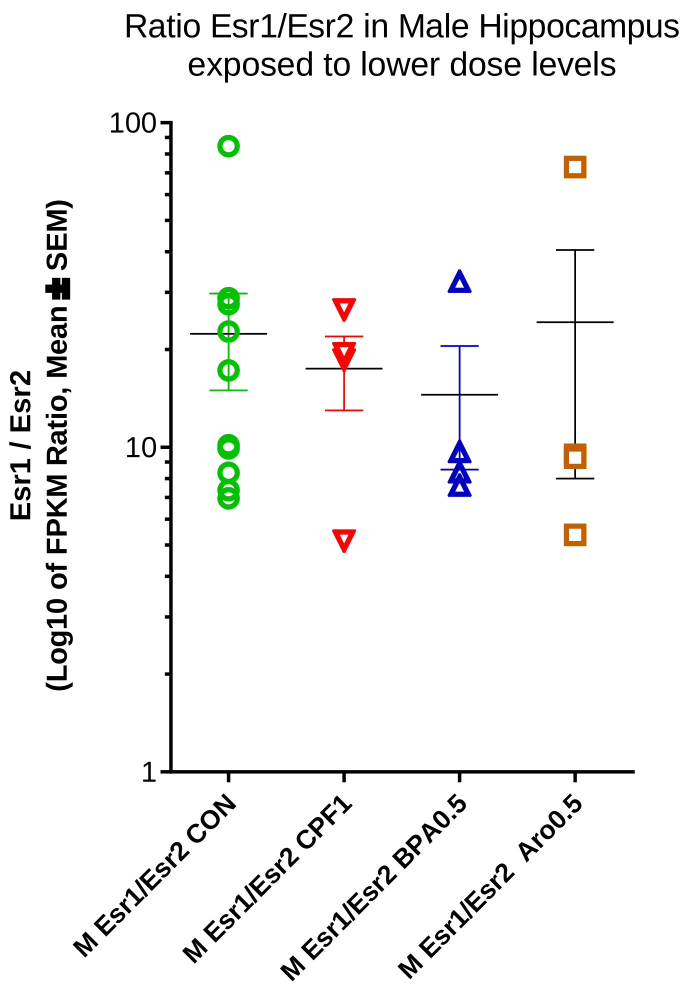

Supplemental Fig. 5. Ratio of ER-alpha (Esr1)/ER-beta (Esr2) in hippocampus of male offspring in the lower dose groups, Aroclor 1254, 0.5 mg/kg (Aro0.5), bisphenol A, 0.5 mg/kg (BPA0.5), and chlorpyrifos, 1 mg/kg (CPF0.1). Transcriptomics analysis. FPKM values of the low-dose groups exhibited a large variability and are shown in logarithmic scale.
